# Viral dynamics in a high-rate algal pond reveals a burst of *Phycodnaviridae* diversity correlated with episodic algal mortality

**DOI:** 10.1128/mbio.02803-24

**Published:** 2024-11-12

**Authors:** E. E. Chase, T. Pitot, S. Bouchard, S. Triplet, C. Przybyla, A. Gobet, C. Desnues, G. Blanc

**Affiliations:** 1 Microbiologie Environnementale Biotechnologie, Institut Méditerranéen d'Océanologie, Campus de Luminy, Marseille, France; 2 Institut hospitalo-universitaire (IHU) Méditerranée infection, Marseille, France; 3 Department of Microbiology, University of Tennessee Knoxville, Knoxville, Tennessee, USA; 4 Department of Biochemistry, Microbiology and Bioinformatics, Université Laval, Québec, Québec, Canada; 5 MARBEC, Univ Montpellier, CNRS, Ifremer, IRD, Montpellier, France; Virginia Tech, Blacksburg, Virginia, USA; Virginia Polytechnic Institute and State University, Blacksburg, Virginia, USA

**Keywords:** *Nucleocytoviricota*, polinton-like viruses, virophage, algal bloom, host-virus dynamics, industrial microalgal cultivation

## Abstract

This study explores virus-host dynamics in a unique environment: an industrial high-rate algal pond (HRAP). A wealth of novel DNA algal viruses are revealed, including members of *Nucleocytoviricota* “giant viruses” and the enigmatic *Preplasmiviricota* (e.g., virophages and polinton-like viruses). Several species of single-celled eukaryotic photosynthetic algae are identified (Chlorophyta) as putative hosts, with alternating dominant populations during the year of study. We specifically observe a surprising diversity of giant viruses from the family *Phycodnaviridae* (*Nucleocytoviricota*), including phylogenetically related but highly diversified genotypes appearing in the HRAP that we suggest are implicated in bloom collapse. We hypothesize that these related *Phycodnaviridae* lineages infect the same algal species of the genus *Picochlorum* that has been identified in the HRAP. This study establishes a baseline for comprehending the role viruses play in algal farming and emphasizes the necessity of controlling the viral load in future culture system development to optimize algal growth.

**IMPORTANCE:**

The virosphere is ubiquitous, but we have yet to characterize many environments where viruses exist. In an industrial polyculture of microalgae, a wealth of viruses persist, their diversity and dynamics changing over time and consequently give evidence of their evolution and ecological strategies. Several notable infectious agents of the culture’s algae appear, including giant viruses, polinton-like viruses, and a virophage. As our reliance and interest in algal compound-based cosmetics, pharmaceuticals, and bio-plastics increases, so must our understanding of these systems, including the unique viruses that appear there.

## INTRODUCTION

The importance of viruses in aquatic systems is well documented from nutrient cycling and algal bloom control in the open ocean (1, 2), to modulating microbial communities in the deepest ocean (3), and throughout the open ocean water column (4). In relation to microalgae, Phylum *Nucleocytoviricota,* which comprises the so-called “giant viruses,” virophages (*Preplasmiviricota*), and polinton-like viruses (*Preplasmiviricota*) represent potential bloom control viruses of interest (e.g., ecological dynamics) and are currently understudied. Family *Phycodnaviridae* and family *Mimiviridae* are two taxa within *Nucleocytoviricota* (Class *Megaviricetes*) known to infect microalgae (5, 6). Virophages are a group of viruses infecting *Nucleocytoviricota* in a tripartite/co-infection system between a giant virus and its host (7). Previous work on virophages has given evidence for potential *Phycodnaviridae* hosts (8) and recently characterized this interaction *in situ* (9). Despite evidence of a virophage benefiting the cellular host indirectly by reducing *Nucleocytoviricota* production in co-infection (10, 11) and regulating host-virus dynamics (8), much is still unknown about these viruses. Polintons (or Mavericks) are relatively large (up to 40 kb) DNA transposons and have been found in a substantial diversity of multicellular and unicellular species, including protists, fungi, beetles, fish, chicken, nematodes, etc. (12). In 2015, a 31 kb DNA virus (i.e.*,* TsV-N1), similar in structure to polintons and with which it shares a number of phylogenetically related genes (13), was identified as viral particles infecting the microalga *Tetraselmis striata* (14). This confirmed that some polinton-like viruses (PLVs) behaved as genuine viruses (i.e.*,* having virions) and not just transposable elements (15). Notably, the PLVs have some differences from polintons, including the absence of specific genes, in particular the maturation protease and retrovirus-type integrase (see reference [16]). Previous metagenomic work concluded that there are at least eight major groups of PLVs and also uncovered many associated with microalgae (17), and a recent meta-study has proposed formal taxonomic designations for both PLVs and virophages (18). Virophages can possess similarities to PLVs (15) and it was suggested that PLVs have the potential to co-infect algae with *Megaviricetes* species (16). Recently, a PLV (Gezel-14T) was isolated and found to contribute to viral-host dynamics in a tripartite system as well (19).

Given the significance of viruses in major aquatic systems, our study focuses on a specific system that is relatively unexplored until now; the DNA viral community of an industrial high-rate algal pond (HRAP). Previously, the RNA viruses were assessed (20). The said HRAP hosts a non-specific polyculture of microalgae sourced from seawater of the Mediterranean Sea. Our objectives were to understand the diversity of microalgal viruses in the system alongside their dynamics during a culturing period. More specifically, the HRAP environment permitted a dense microalgal culture not unlike an algal bloom of a natural aquatic environment, consequently providing an opportunity to investigate a bloom-like situation and the viral community during this period. Each of the aforementioned virus groups (i.e., orders *Algavirales* and *Imitervirales* [including families *Phycodnaviridae* and *Mimiviridae*], and Phylum *Preplasmiviricota* [virophages and PLVs]) were investigated within the microalgae culturing HRAP of this study to gain an understanding of their role in a dense culture that we suggest is in many ways analogous to a natural microalgal bloom. Furthermore, these groups are among the most enigmatic and evolutionarily interesting DNA viruses.

## MATERIALS AND METHODS

### Sampling, filtration, nucleic acid extraction, and next-generation sequencing of HRAP-sampled water

Water sampling and HRAP characteristics are described in a previous publication (20). Briefly, water sampling was conducted from a 160 m^2^ race-way style open-air basin in Palavas-les-flots, France, which occasionally experienced culture “crashes” (i.e.*,* a die-off of algae). A non-specific algal inoculant was collected from the Mediterranean Sea (Plage du Prévost) to supply the system. Samples were processed (e.g.*,* centrifuged, filtered) in the same manner to produce an “ultravirome” of “small” DNA viruses (i.e., <0.2 µm), without the need for the production of a cDNA library as in our previous analysis of RNA viruses. As an attempt to isolate and subsequently sequence *Nucleocytoviricota*, fluorescence-activated cell sorting (FACS) was carried out in the size fraction 0.2 μm–1.2 µm labeled using SYBR-Green and analyzed according to the SSC (side scatter) and FITC (Fluorescein Isothiocyanate) fluorescence parameters. The size fraction was obtained by filtering samples through a 1.2-µm pore filter (Sartorius ref:17593), then concentrated 25× on a tangential flow filtration column with a nominal pore size of 0.2 µm (Microkros ref: C02-P20U-05-N). Four gated populations, “megaviromes,” corresponding to areas of the SSC-FITC plot (where giant viruses are generally observed [21]) were sorted in each sample (Fig. S1). Note that some bacterial populations may emerge in the same area, thus come to contaminate the megaviromes. The number of isolated particles (events) per population ranged between 89,000 and 500,000. Lysis was carried out as detailed in reference (22), and nucleic acid was extracted using EZ1 Advanced XL extraction with a Virus Card (QIAGEN). Six of the 20 FACS populations did not generate sufficient DNA and were not subsequently sequenced. Library preparation and paired-end 2 × 251 bp Illumina MiSeq sequencing of the ultraviromes were done as described in a previous publication (20). FACS populations were amplified using a Ready-To-Go Genomiphi V3 kit (GE Healthcare), followed by purification and Illumina HiSeq sequencing (pair-end 2 × 151 bp) performed by DOE Joint Genome Institute (JGI). In addition, a pooled sample of DNA ultraviromes (DNAUV) with roughly equal concentrations of each sample’s nucleic acids from each month was also sequenced by JGI in the same way for increased sequencing coverage.

### Quality control and contig assembly of metagenomes, taxonomic assignments, and functional annotation

Quality control steps with raw reads were as described in previous work (20). Assemblies of ultraviromes and megaviromes were done with SPAdes using the metaviral algorithm and the single-cell algorithm, respectively (23). Assembly statistics were done using QUAST (24) and are reported in Table S1. Taxonomic assignments were given by an “open reading frames (ORFs) voting” procedure. To fulfill this, ORFs greater than 100 codons were extracted from each contig using EMBOSS getorf (25) with default parameters in the ultraviromes and megaviromes, aligned against TrEMBL (26) using MMseqs2 (27) with e-value <10^−05^ and the taxonomic information of the ORF best hits were recorded. Contig taxonomic classification was done using a majority rule criterion over the ORF best hits. In total, 1,977 contigs were classified as viral within the megaviromes (1,398 eukaryotic viruses, 576 prokaryotic viruses, and 3 unknown host viruses) and 1,114 within the ultravirome contigs (62 eukaryotic viruses, 1,037 prokaryotic viruses, and 15 unknown host viruses) (see Table S2). The DNAUV assembly contained 1,288 putative viral contigs, with a categorical distribution similar to that of the combined ultravirome assemblies (47 eukaryotic viruses, 1,235 prokaryotic viruses, and 6 unknown host viruses). Information on the putative function of ORF-encoded proteins in viral contigs was searched among the top hits found by MMseqs2 in various protein sequence databases, including Swissprot, COG (Cluster of Orthologous Genes), VOGDB (Virus Orthologous Groups), and Uniref100.

### Identifying potential microalgae hosts using 18S rDNA metabarcoding of HRAP water

To characterize the diversity of eukaryotic microorganisms by metabarcoding, DNA was extracted from 0.2 µm pore-sized filters after sample water filtration using a DNeasy PowerWater Kit (QIAGEN) based on the manufacturer’s instructions, and the V4 variable region of the 18S ribosomal RNA gene was amplified using eukaryote-specific universal primers (28). The obtained amplicons were then subcontracted by the Genotoul facility (Toulouse; get.genotoul.fr) which performed indexing and Illumina MiSeq sequencing. The sense and antisense sequences obtained were assembled and then clustered in the form of an amplicon sequence variant (ASV) and identified at different taxonomic levels using the PR2 database (29). These analyses were performed under R software version 3.6.2 using the *dada2* package (30). Raw sequences were deposited at the National Center for Biotechnology Information (NCBI) under the BioProject identifier PRJNA1098217.

### Phylogenetic construction of phyla *Nucleocytoviricota* and *Preplasmiviricota*

A major capsid protein (MCP), DNA polymerase B (PolB), and ATPase tree were produced using publicly available data for *Nucleocytoviricota* using a multiple-sequence alignment from MAFFT v.7 (31), and FastTree (32) default settings. In addition, a polinton-like virus tree (MCP based using publicly available data) was composed also using FastTree (32) default settings. MCP sequences were downloaded from the National Centre for Biotechnology Information (NCBI) for all virophages. Corresponding sequences were also aligned using MAFFT v.7 and the virophage tree was produced using FastTree with default settings. All trees were run with 1,000 bootstrap replicates. In all cases, putative sequences from relevant groups recovered from the HRAP were included in the trees. Note that these trees alone cannot produce the best hypothesis of *Nucleocytoviricota* but provide context for the diversity of the HRAP (PolB, ATPase), and the putative virus contigs discussed herein (MCP).

### Phylogenetic construction of horizontal gene transfer candidate genes

To create phylogenetic trees of horizontal gene transfer candidates, homologs of target HRAP viral proteins were searched in the NCBI Refseq protein database using BLASTP (e-value <0.05). To limit sequence redundancy among homologous proteins, the 100 top-scoring Refseq hits were clustered using CD-HIT (33) with a sequence identity criterion of −c = 0.75. The clustered proteins were then aligned with the query viral proteins using MAFFT (31). The resulting multiple-sequence alignment was fed into FastTree (32), which ran with the default settings. All tree reconstructions were run with 1,000 bootstrap replicates.

### Dynamics of putative viral targets by qPCR

The selection of putative viruses for targeting, processing of raw water samples for qPCR, and the qPCR reaction setup were conducted in the same way as described in previous work (20) without the need for cDNA construction. Briefly, Primer3 (34) and PerlPrimer v.1.2.3 (35) were used to produce efficient primers *in silico* before *in vitro* testing, see Data S1 for primer sequences and additional information. All primer sets were designed to amplify MCP gene targets. The number of reaction cycles (45) minus the Cq value was reported as the “inverted Cq.”

### Using correlation among viruses and potential hosts to uncover possible host-virus interactions

Principal component analyses (PCA) were performed and visualized on a combined data set of standardized metabarcoding (18S rDNA) and qPCR results using R package *factoextra*, using time points where both metabarcoding and qPCR results were available. Similarity profile analysis (SIMPROF) hierarchical clustering at α = 0.1 was conducted using R package *clustsig*, and correlation networks were visualized using Cytoscape v3.9.1 (36). Publicly available sequenced alga of potential hosts were checked for recent *Nucleocytoviricota* viral insertions using ViralRecall (37).

## RESULTS AND DISCUSSION

### Metagenomic sequencing data and biodiversity of putative DNA viruses in the HRAP

In total, five ultraviromes from different months of 2018 (i.e., April [17.04.UV], May [17.05.UV], July [05.07.UV], September [11.09.UV], and October [23.10.UV]) were sequenced, and 16 populations based on FACS gated populations (see Table S1). In addition, a pooled sample of the five DNA ultraviromes was also sequenced for increased sequencing coverage. Following genome assembly, the number of contigs greater than 2 kb ranged from 74 to 9,107 for the ultravirome samples, and 740 to 10,045 for the megaviromes, with the average number per sample being 2,536 and 3,436 for the ultravirome and megaviromes, respectively. The average GC content in the ultraviromes ranged from 50% to 55%, whereas in the megaviromes it ranged from 38% to 53%. Bacterial sequences were abundant in all viromes (Fig. S2; Table S2); however, virus contigs still represented between 0.3% (17.05.P4) and 30.1% (05.07.UV) of the data. As our taxonomic assignment method is based on the analysis of best hits, these percentages are likely to be underestimated due to the existence of proviruses inserted into the genomes of their host cellular organisms. Thus, viral contigs whose closest relative is a provirus will tend to be classified as a cellular organism rather than a virus. Eukaryotic virus sequences were generally dominant (in terms of contig cumulated lengths) over prokaryotic virus sequences in megaviromes (the mean proportion was 4.9% for eukaryotic viruses versus 1.4% for prokaryotic viruses) while this trend was opposite in the ultraviromes (mean frequency was 1.8% versus 14.6%, respectively). The proportion of identified ssDNA virus sequences was generally very low, totaling 0.14% of all viral sequences in megaviromes and 1.12% in ultraviromes.

**Fig 1:**
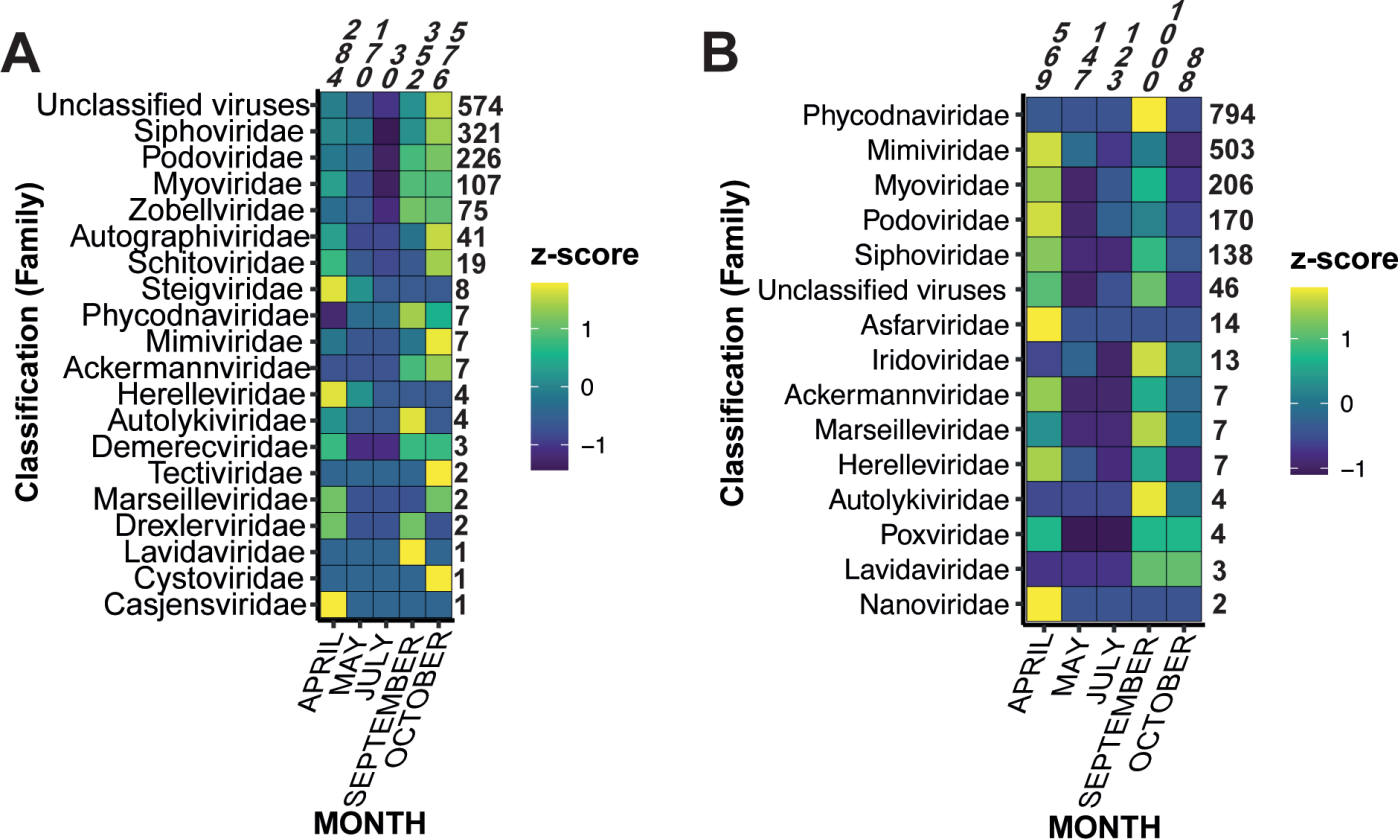
Proportion (by z-score) of viral taxonomic assignments to assembled (**A**) DNA ultravirome contigs by each metagenomic sample in 2018, and (**B**) proportion of viral taxonomic assignments to assembled metagenomes produced from FACS population, grouped together by sampling month. Please note (**B**) is only to showcase the diversity and is not a quantitative assessment as some sample months produced more FACS populations of interest, and therefore attributed more sequencing. In addition, these classifications result from our “ORF voting” procedure. The total numbers of contigs attributed to each family is shown on the y-axis, and the total number of contigs attributed to each month is shown on the x-axis.

Using metagenomics, we employed two approaches to uncover DNA viruses of the HRAP, permitting the observation of small DNA viruses within an ultravirome, and large DNA viruses (e.g., *Nucleocytoviricota*) through FACS populations and further processing. The ultraviromes are primarily composed of phages (i.e., class *Caudoviricetes*), and unclassified viruses (Fig. 1A). Polinton-like viruses were also uncovered in the DNA ultraviromes and megaviromes (see phylogeny in Fig. S3) and are discussed more below. Of special importance to our study is our effort to retrieve *Nucleocytoviricota* by FACS methods, and we were able to recover a viral diversity community composition different than the ultravirome fraction (Fig. 1B). Recent work (38) on metagenomic sample filtration methods points out the almost definite loss of viral community taxonomic information in metagenomic studies where larger (>0.45 µm pore size) water fractions are removed and left un-sequenced. No doubt, sequencing this large viral fraction permitted higher recovery of *Nucleocytoviricota* compared to the ultravirome method. A putative member virophage was recovered and does not group closely with any characterized viruses (Fig. S4). Virophages are found in association with *Phycodnaviridae* (39) and *Mimiviridae* (40), both of which were recovered in our megavirome samples (Fig. 1B) and will be explored in more depth.

### Coding capacity recovered from identified viral contigs

Gene prediction and functional annotation of contigs assigned to eukaryotic and prokaryotic viruses were 4,292 and 10,237 ORFs with a significant MMseqs2 match in Uniref100. The bulk of these ORFs were homologous to genes already found in viruses (Figure S5 A and B; Table S3) since 67% and 69% of ORFs from eukaryotic and prokaryotic viruses, respectively, had viral top hits. In addition, respectively, 27% and 28% of ORFs had non-viral top hits but still matched viral homologs in subsequent hits. Finally, 260 ORFs (6%) were carried by eukaryotic virus contigs, and 360 ORFs (4%) were carried by prokaryotic viruses aligned exclusively with sequences from cellular organisms. These ORFs are particularly interesting because they could originate *via* horizontal gene transfer, which has not been observed before with these genes. A selection of them is discussed below.

A fraction of the ORFs (46% and 32% of eukaryotic and prokaryotic viruses, respectively) could be assigned to clusters of orthologous genes (COG and VOG), from which we derived a classification in functional categories (Figures S5 C and D; Table S3). ORFs encoding typical viral functions were broadly grouped into the categories “virus replication,” “virus structure,” and “mobilome.” The latter category, defined in the COG database scheme, essentially comprised prophage-related genes in our data set. Overall, these three categories represented 27% and 40% of ORFs in the eukaryotic and prokaryotic viruses, respectively. Categories “information storage and processing” (28% *vs*. 26%) and “metabolism” were relatively equivalent (17%) in frequency between eukaryotic and prokaryotic viruses. The “metabolism” category contained numerous ORFs that potentially fall within the definition of auxiliary metabolic genes (AMGs), that is*,* genes of cellular origin encoded by viruses that regulate host cell metabolism during infection, enabling the virus to replicate more efficiently (41). By contrast, the “cellular processes and signaling” category was more prevalent in eukaryotic viruses (27%) than in prokaryotic viruses (17%), especially for functions involved in post-translational modification, protein turnover and chaperones (9% vs. 2%, respectively) as well as signal transduction mechanisms (6% vs. 3%). This difference may reflect a more complex protein maturation process and a more elaborate regulation of host metabolism by the eukaryotic viruses found in our metagenomes, which are primarily members of *Nucleocytoviricota*.

Several genes found in eukaryotic virus contigs exhibiting matches solely in cellular organisms are homologs to proteins with relatively well-defined cellular functions. Of particular note are proteins listed in Table 1 such as cobalt transporter, iron transporter, Calvin cycle protein CP12, heme oxygenase 1, phycocyanobilin:ferredoxin oxidoreductase, photosystem I assembly protein Ycf3, and phosphatidylserine decarboxylase, which, until now, have remained undocumented within eukaryotic viral genomes. Their corresponding genes were all identified within contigs assembled from September megaviromes and were consistently flanked by other genes of viral origin (i.e.*,* with viral best hits), some of which code for proteins characteristic of *Nucleocytoviricota*, including MCP, DNA packaging ATPase, and very late transcription factor 3 (VLTF3). The listed genes’ best match was in bacteria, cyanobacteria, or diatoms, indicating their putative origin of the horizontal gene transfer. Cobalt is a key component of certain enzymes, including coenzymes such as cobalamin (vitamin B12), involved in the biosynthesis of nucleic acids and amino acids (42). This trace element is also a component of proteins involved in metal transport, giving it a crucial role in nutrient uptake and utilization by cells. Iron is an essential component of chlorophyll and is involved in cellular respiration and nitrogen fixation (43). The identified transporter genes could enable viruses to increase the host’s capacity to acquire these essential nutrients from the environment during infection to boost the host’s metabolism. Calvin cycle protein CP12 is known to play a key role in dark/light regulation of the Calvin-Benson-Bassham (CBB) cycle responsible for CO_2_ assimilation and has been associated with several other putative functions, including algal CO_2_-concentrating mechanisms and oxidative stress (44). The heme oxygenase 1 and phycocyanobilin:ferredoxin oxidoreductase are both involved in a pathway that produces pigments in the algal chloroplast with important regulatory functions: increasing chlorophyll synthesis, signaling the nucleus to produce antioxidants, and stabilizing Photosystem I (45, 46). The Photosystem I assembly protein Ycf3 is essential for the assembly, stability, and optimal functioning of Photosystem I in algae (47). The phosphatidylserine decarboxylase catalyzes the conversion of phosphatidylserine into phosphatidylethanolamine, an essential phospholipid for maintaining membrane integrity and fluidity (48). The listed genes’ closest phylogenetic neighbors were in bacteria, cyanobacteria, or photosynthetic eukaryotes, indicating the putative origin of the horizontal gene transfer (Fig. S6). Overall, these novel viral proteins play a variety of roles in cellular processes such as nutrient transport, photosynthesis, pigment synthesis, and membrane biogenesis, underscoring the importance of specifically understanding viral interactions with algae in the HRAP ecosystem.

**TABLE 1:** Selection of genes from eukaryotic virus contigs representing novel findings and likely horizontal gene transfer origins

Protein function	Putative origin^*a*^	Viral evidence	
		VBM/ORFs^*b*^	Viral hallmark proteins^*c*^
Cobalt transporter	Bacteria	4/6	
Iron transporter	Bacteria	17/25	VLTF3
Calvin cycle protein CP12	Cyanobacteria	4/8	MCP
Heme oxygenase 1	Ochrophyte	3/5	ATPase
Phycocyanobilin:ferredoxin oxidoreductase	Chlorophyta	4/6	MCP
Photosystem I assembly protein Ycf3	Cyanobacteria	4/7	
Phosphatidylserine decarboxylase	Bacteria	25/27	MCP, VLTF3

^*a*^ Based on the closest phylogenetic neighbor (see Fig. S6).

^*b*^ Number of ORFs with viral best match/total number of ORFs within contig.

^*c*^ Viral hallmark proteins encoded by surrounding ORFs.

### Biodiversity of potential alga hosts in the HRAP

**Fig 2:**
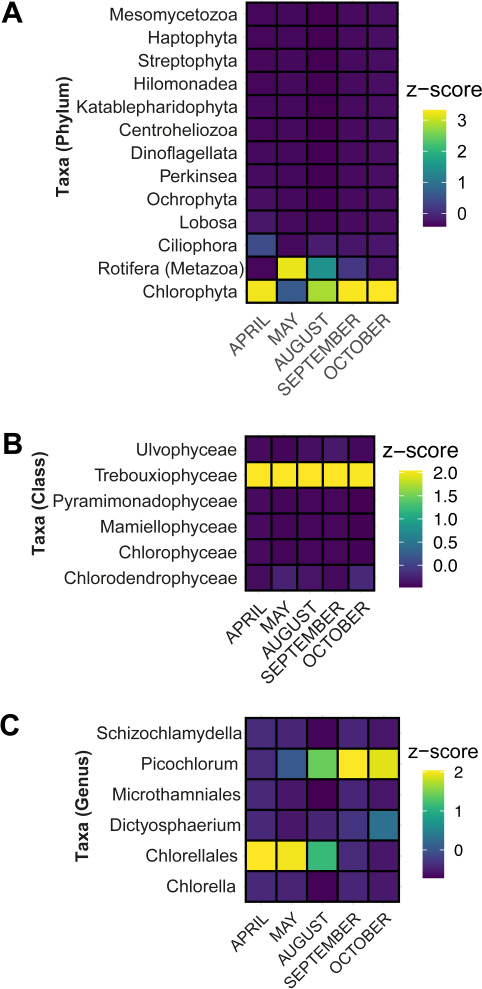
Classification and prominence of HRAP eukaryotic microbes of interest (i.e., possible virus hosts) and an ecologically important microfauna (rotifera). (**A**) Major groupings of algae (primarily phylums) and one metazoan (Rotifera), (**B**) the phylum Chlorophyta is explored more based on class given its importance in the HRAP, and (**C**) genera present at the basin within class *Trebouxiophyceae*.

Herein we focused primarily on possible hosts of our recovered virus sequences; photosynthetic single-celled eukaryotes. The most abundant taxa are class *Trebouxiophyceae* (Chlorophyta), with phylum Rotifera also being important in the HRAP (Fig. 2A). Rotifers are well-documented grazers of microalgae that have been shown to reduce biomass production in open-faced microalgae cultures (49) (e.g.*,* HRAPs). However, they are not present at all culture die-off points throughout the time series and are not the focus of our study (50–53). Of specific interest to our study are several groups of unicellular microalgae that are present (Fig. 2B). Although in smaller abundances than *Trebouxiophyceae, Ulvophyceae, Pyramimonadophyceae, Mamiellophyceae, Chlorophyceae,* and *Chlorodendrophyceae* appear in the HRAP during the 2018 HRAP samples. Furthermore, within class *Trebouxiophyceae*, *Picochlorum,* and another member of order *Chlorellales* (unidentified genus) are abundant (Fig. 2C). Given previously established associations with DNA viruses to division Chlorophyta (54, 55), we focused mostly on these microalgae and not additional unicellular plankton identified in the HRAP by metabarcoding, as these species do not appear in substantial amounts (based on ASVs) in 2018 alongside our metagenomic study or present evidence of relationships with the putative viruses tracked in our study (see Table S4).

### Predicting potential microalgal hosts and their viruses

Using a correlation of microalgal species and viral population presence/relative abundance, we can try to resolve potential host and virus relationships (56, 57). As the approach is simply observing correlations, it cannot account for ecological nuances of host-virus relationships, namely it assumes that overlapping host and virus presence and their relative abundances are indicators of infection. With this understanding, we interpreted our results as a preliminary suggestion of possible host-virus relationships in the context of known viral ecology. Correlation analyses were inconclusive for virus-host relationships outside of Chlorophyta alga, and consequently, we focused solely on this group.

Principal component analysis (PCA) with members of families *Mimiviridae, Phycodnaviridae,* and a virophage (sequenced within megaviromes) showed a strong positive correlation (i.e., higher covariance) among two *Picochlorum spp*. and a member of *Chlorellales* with several members of these viruses (Fig. S7A), including a putative phycodnavirus that also had a strong correlation with the putative virophage (see Table S5 for correlation coefficient output). A strong correlation was shown between a member of the family *Mimiviridae* and the alga group *Dictyosphaerium* (correlation coefficient = 0.8687, Fig. S7A). These relationships are primarily associated with September and October (2018) sampling. Although much less supported by these principal components, there was an additional association between a member of the family *Mimiviridae* and an unknown species from the order *Chlorellales* (correlation coefficient = 0.7372). The PCA for polinton-like viruses (PLVs) does not show a close correlation for some of the putative viruses (Fig. S7B). Some PLVs are associated with the genera *Picochlorum* and *Tetraselmis*, and to some extent the genus *Dictyosphaerium*. Hierarchical clustering indicates a significant grouping among previously described megavirome viruses and hosts (Fig. S8; groups b, d, and e). In addition, some PLVs and their associated PCA-suggested hosts also produce significant groupings (Fig. S9). Correlations among both the megaviromes and PLVs with genus *Picochlorum* reflect an apparent bloom of *Picochlorum* in the area where the HRAP is stationed during these months; however, in comparison to the PLVs, the correlation among 11 *Phycodnaviridae*, one *Mimiviridae*, and the putative virophage are specifically much stronger (Fig. S7B). In the context of viral ecology, it seems more likely that correlations found among *Nucleocytoviricota* and a virophage could imply potential hosts more readily than PLV and alga correlations. We attribute this to the likely rapid proliferation of the *Nucleocytoviricota* groups after infection (e.g., *Chlorella* virocells release PBCV-1 viral particles 3 hours post-infection [58]). In the case of PLVs, although relatively little is known, their dual lifestyle (16, 59) of insertion, and later proliferation may require specific cues for a virocell to develop and release viral particles effectively uncoupling infection and release of viruses acutely, thereby confounding the interpretation of correlation analysis. Although virophages could be uncoupled in the same way due to viral integration capabilities, in this case, a known trigger for viral particle production is present (e.g., co-infection with *Nucleocytoviricota* [11]) and conclusively we expect all members of this tripartite infection to co-exist when virophages are detected in the HRAP. In this scenario, detection of the putative virophage indicates viral particles being produced and this would be in response to cellular hosts with a *Nucleocytoviricota* infection taking place (i.e.*,* a virocell is formed). With ecology in mind, we expect to be able to produce some valid indication of both *Nucleocytoviricota* and virophage alga hosts through correlation analyses; however, PLV hosts’ indications are less straightforward. In addition, some interactions could be occurring between PLVs and *Nucleocytoviricota*, but our PCA and hierarchical clustering analyses did not produce clear interactions (See Fig. S10); in one instance, several host species (*Tetraselmis* spp. and *Dictyosphaerium*) cluster with a putative *Nucleocytoviricota* (M1) and three PLVs (2, 4, 5) but given the number of hosts within the cluster (four total), we were unable to support the likelihood of a *Nucleocytoviricota*-PLV and host tripartite infection in this context.

**Fig 3:**
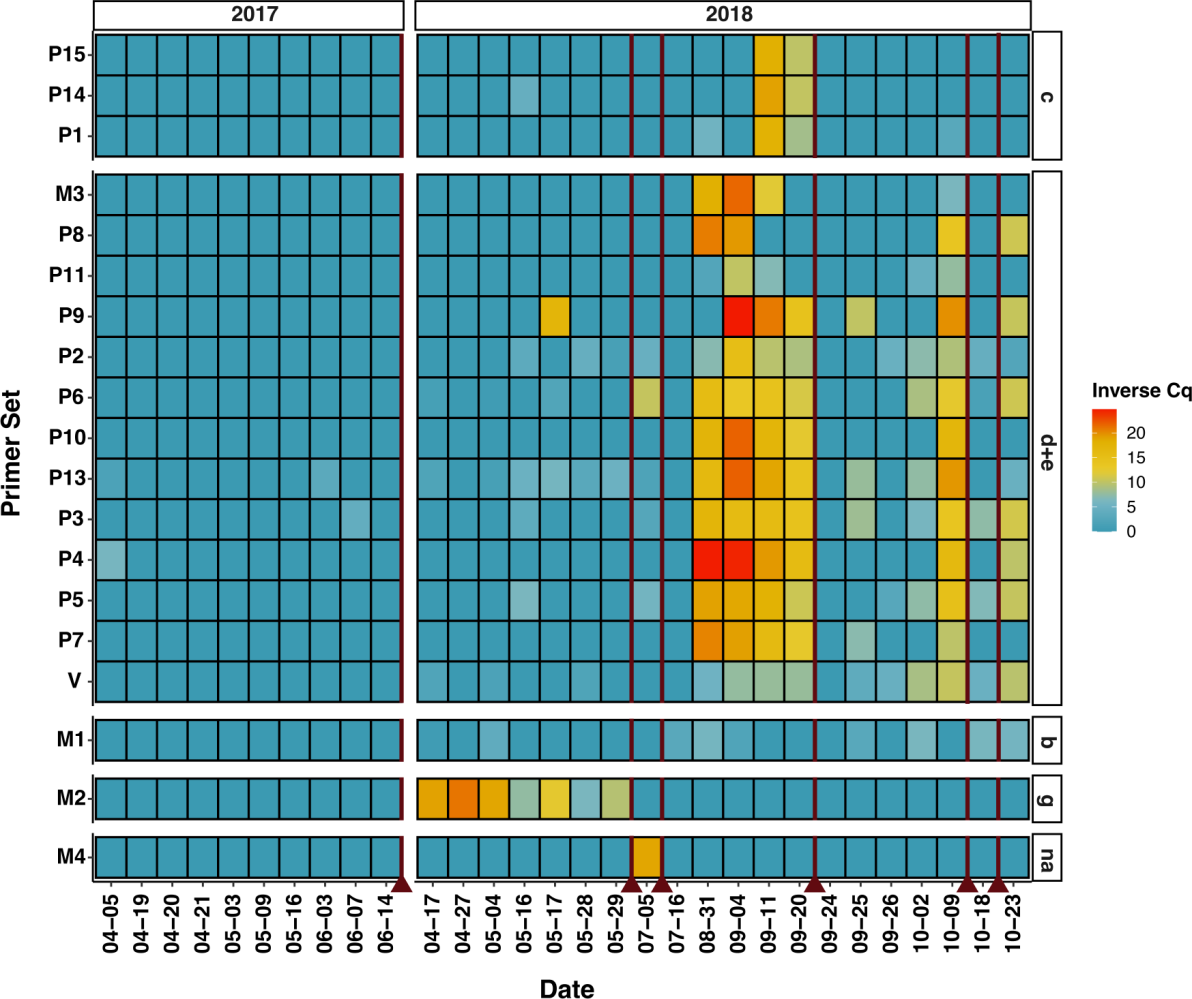
Putative *Nucleocytoviricota* and virophage tracking by qPCR throughout 2017 and 2018 samples. V, P, and M indicate potential virophage, family *Phycodnaviridae*, and family *Mimiviridae* respectively. An inverse Cq is calculated by the number of cycles (total 45) minus the mean Cq value across technical triplicate reactions for each instance (e.g., viral target and sample data). Redlines indicate HRAP crash dates.

**Fig 4:**
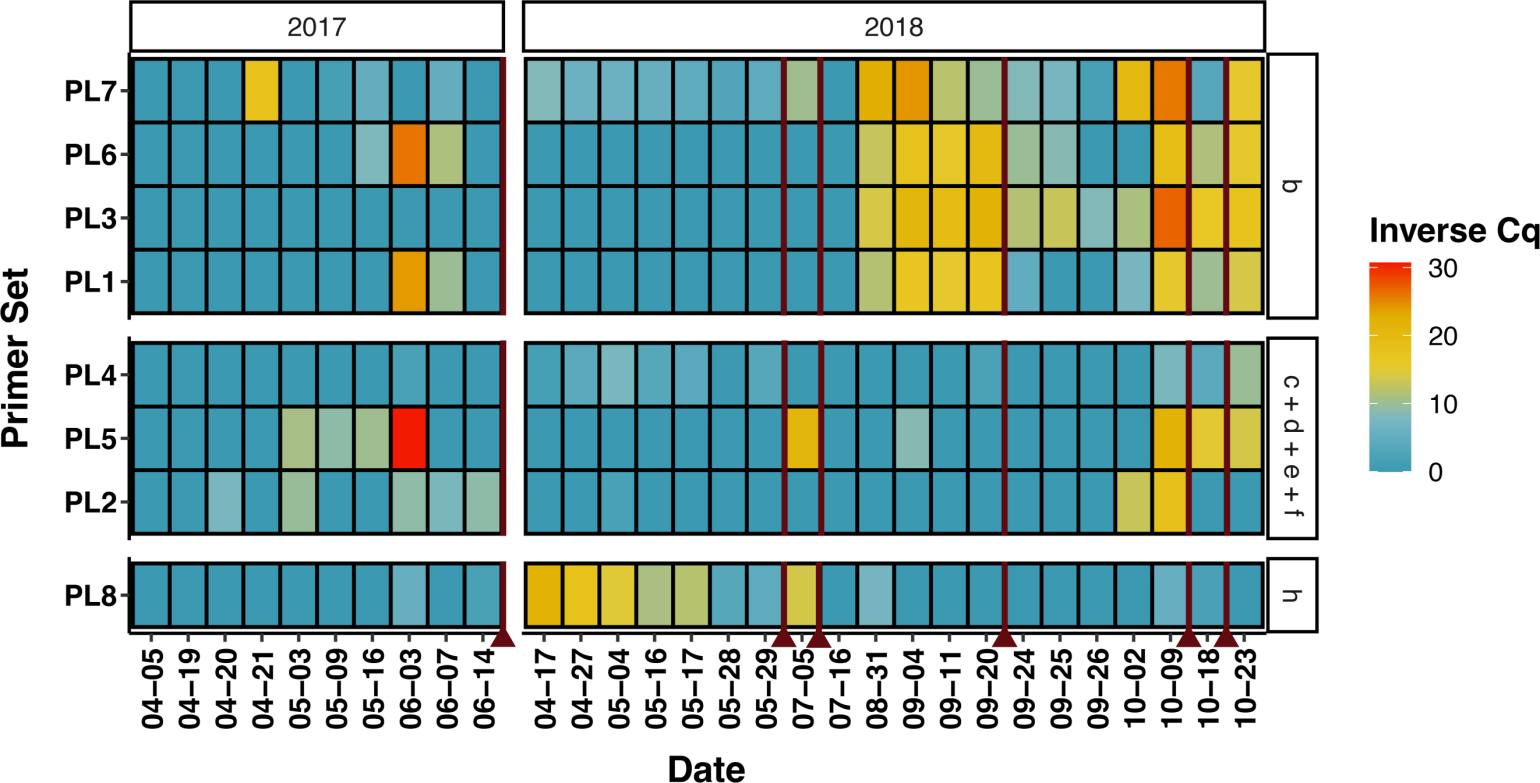
Putative polinton-like virus (PLV) tracking by qPCR throughout 2017 and 2018 samples. PLV indicates potential polinton-like viruses. An inverse Cq is calculated by the number of cycles (total 45) minus the mean Cq value across technical triplicate reactions for each instance (e.g., viral target and sample data). Redlines indicate basin crash dates.

**Fig 5:**
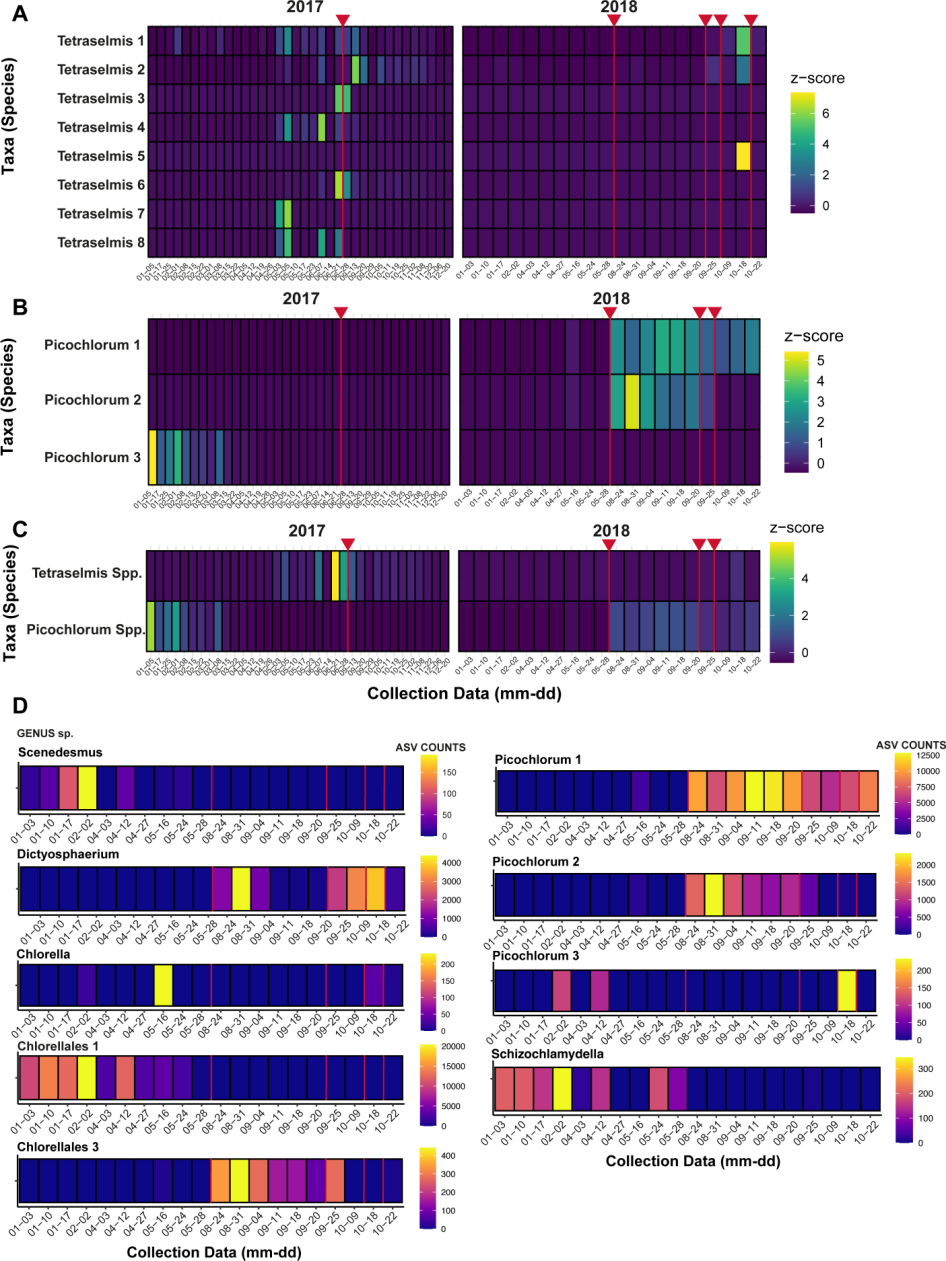
Presence and relative amounts (based on ASVs of 18S rDNA data) of potential microalgae hosts using 2017 and 2018 data (except in the case of **D**) collected by water samples of the basin system. The diversity of two highly abundant potential hosts is depicted, (**A**) *Tetraselmis* spp. and (**B**) *Picochlorum* spp., alongside their overall relative abundance of the HRAP over time (**C**). (**D**) The most abundant potential microalgal hosts during 2018 at the genus level are also shown, including the individual *Picochlorum* spp. that were important to 2018. ASV counts are indicated by color legends. Instances where a new culture run is made, after a die-off, are indicated by a red line.

Using our full qPCR data set (Fig. 3 and 4) and Chlorophyta metabarcoding results (Fig. 5), we can further hypothesize virus-host interactions using our correlation and clustering work as an initial indication of potential hosts. Overall viruses within hierarchically clustered groupings exhibited similar patterns of presence and absence based on qPCR results in both *Nucleocytoviricota* and the putative virophage (Fig. 3) and PLV analyses (Fig. 4). Across 2017 and across 2018, *Tetraselmis spp*. and *Picochlorum spp*. dominate the basin (Fig. 5A through C). Herein, we focus primarily on the 2018 dynamics given that our metagenomic samples were collected then. Notably, the *Nucleocytoviricota* and virophage grouping (Fig. S8D) reflect *Picochlorum* 1 and 2 ASVs quite closely (Fig. 5D), strengthening our hypothesis that members from this grouping could be infecting members of genus *Picochlorum*. Finally, the single *Mimiviridae* (M2) in group G (Fig. S8) has further evidence for infecting *Chlorellales* 1 (Fig. 5D), as previously indicated by our correlation analyses, given both of their nearly complete disappearance after May 2018. Dynamics also overlap between M2 and *Schizochlamydella* and *Scenedesmus*; however, their presence (ASV counts) is much lower than that of *Chlorellales* 1.

Two of the PLVs most closely related to a known PLV infecting *Tetraselmis striata* (Fig. S3; PLV 2, 5) correlate to some degree with *Tetraselmis* spp. in the HRAP based on our PCA analysis (Fig. S7B); however, one does not (PLV 7). We cannot confidently conclude that these tracked PLVs infect *Tetraselmis* spp. given the theoretical decoupling of PLVs and hosts discussed above but also because an important peak of *Tetraselmis* spp. ASVs occur during a period where qPCR data are unavailable (mid-June to autumn 2017; Table S4). This peak would likely be an important part of the data to assess the specific relationship (if any) between PLV 2, 5, and 7 and *Tetraselmis* spp. Several of the PLVs (Fig. 4B) group with *Picochlorum* 1 and their diversity could again reflect the bloom of *Picochlorum* occurring locally. Phylogenetically closely related PLVs could be infecting completely different organisms; therefore, the relationship of PLVs 2, 5, 7, to TsV-N1 (and each other) does not completely confirm infection of the same species. Overall, correlating PLVs and hosts by these methods is made difficult by their ecology (and our minimal understanding of PLVs at present). No other classifications (by ASVs) outside of Chlorophyta correlate significantly (including hierarchical clustering analysis) with these PLVs (data not included), despite the discovery of PLVs infecting several groups outside of Chlorophyta (17). However, alternative hosts (i.e.*,* not members of Chlorophyta) cannot be ruled out by these *in silico* analyses alone. An important point is that viruses found in different taxonomic groups can also infect the same organism (17); consequently, it is difficult to infer a host of these tracked PLVs. Regardless of the true host, this HRAP appears to be an interesting and significant reservoir of PLVs, some likely to be infecting microalgae.

Given the similarity of *Picochlorum* 1 and 2 (Fig. 5) and group d and e viruses (Fig. 3) dynamics (i.e.*,* presence and abundance) with visual inspection and the results of our correlation analyses, the possibility of a previously undocumented infection of *Nucleocytoviricota* and a tripartite infection with a putative virophage to a *Picochlorum* required further investigation. Available *Picochlorum* genome assemblies were downloaded from NCBI to check for *Nucleocytovirivota* integrated *Nucleocytoviricota* genes/viral elements (GenBank accessions GCA011316045, GCA002818215, GCA009650465, GCA000876415). Several regions with *Nucleocytoviricota* signatures were recovered among these genomes (median size of ~15 kb; Table S6). A *Picochlorum* genome recovered from the Mediterranean Sea (Costa Vermeille) (60) possessed regions with putative superfamily II helicase, DNA polymerase B, packaging ATPase, RNA polymerase, and D5 primase-helicase markers. These results suggest that *Nucleocytoviricota* do or have infected *Picochlorum* in the past (recent and/or distant), and specifically in the close geographical area where the HRAP was situated (although we do not suggest these past infections are integrations of the exact phycoviruses recovered from the HRAP).

### Diversity of *Phycodnaviridae* may reflect the diversity of the host

**Fig 6:**
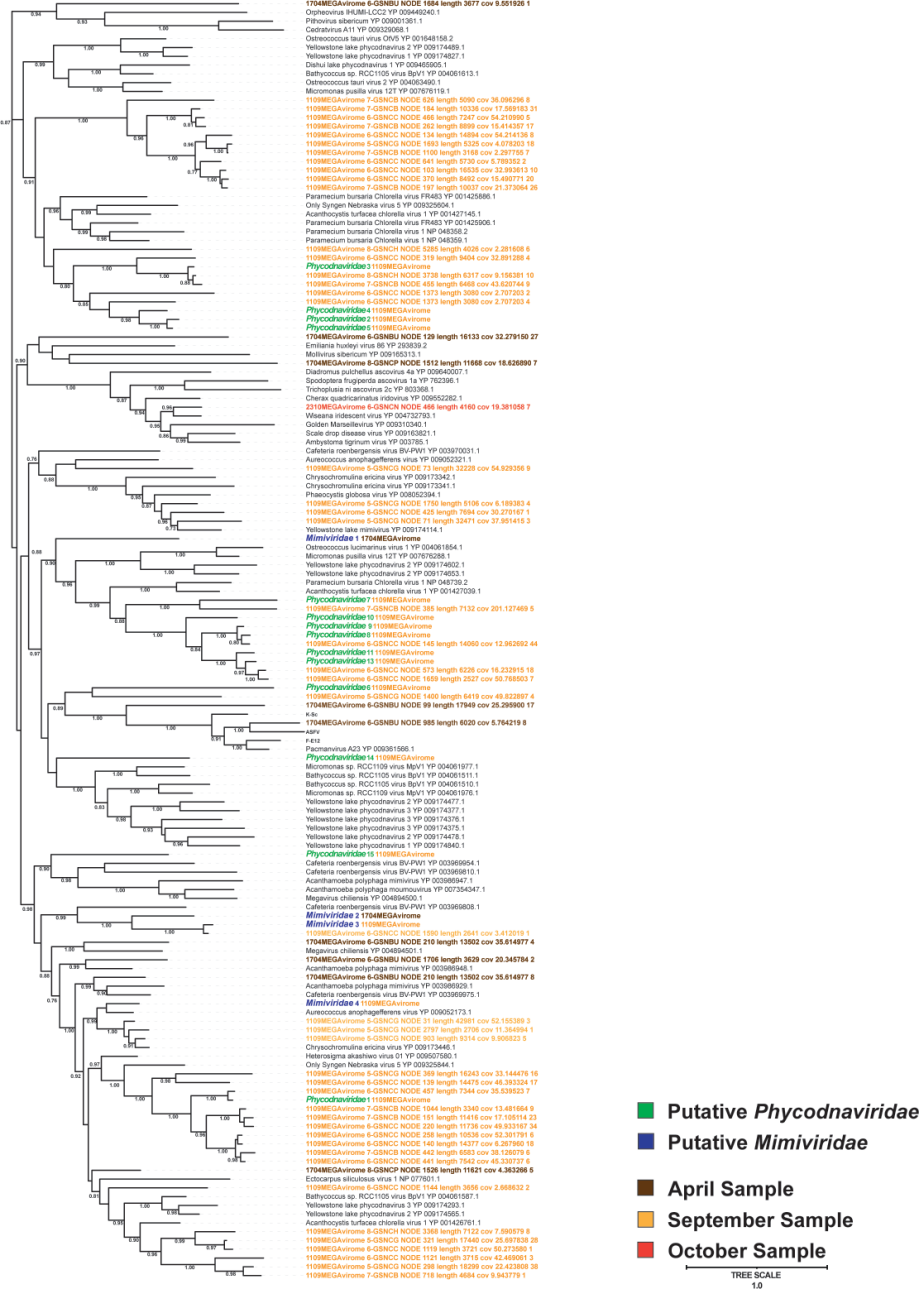
Phylogenetic relationship of *Nucleocytoviricota* found within the study (denoted as “unclassified”), and sequences obtained from NCBI GenBank (accession numbers as indicated) based on major capsid protein (MCP) amino acid sequence. Groups are assigned based on clades of closely related (by sequence similarity) novel *Nucleocytoviricota* for further study. Bootstrap support is indicated and based on 1,000 replicates.

Of substantial interest in this study is the scale of *Phycodnaviridae* (phycodnaviruses) uncovered in our megaviromes. With respect to DNA polymerase (PolB; a relatively robust single-copy gene for phylogenetic reconstruction of *Nucleocytoviricota* [61, 62]), there are a considerable number of putative *Phycodnaviridae* (16 total; Fig. S11). A similar number of packaging ATPase sequences (another marker of *Nucleocytoviricota*) with *Phycodnaviridae* hits are also recovered from the megaviromes (15 total; Fig. S12). *Mimiviridae* representatives also appear in both phylogenetic trees, although in less abundance. It is known that *Phycodnaviridae* genomes of genera *Prasinovirus* and *Chlorovirus* contain multiple copies of the MCP gene (i.e., paralogs) (61); therefore, the sheer number of MCP hits in the corresponding phylogenetic tree (Fig. 6) may be at least partially explained by intragenome duplication. Nonetheless, as a core gene of *Nucleocytoviricota* (63), putative MCPs are a vital finding in the HRAP because they signal true viral hits and this phylogeny contextualizes the *Nucleocytoviricota* MCPs further assessed herein (despite not being the optimum marker for *Nucleocytoviricota* phylogeny for taxonomic purposes).

The HRAP *Phycodnaviridae* included in our group “d” (Fig. S8) appear to form a repertoire of closely related genomes. Given our evidence that they infect the same *Picochlorum* host species, they would have to compete for access to the host. Under competitive conditions, the maintenance of a large diversity of viruses is not the most likely scenario since ecological theory rather predicts the hegemony of the most fit virus. An alternative scenario would be that the different viral lineages have evolved different strain specificity within the same host species. Under the very favorable condition of an HRAP culture, multiple algal strains could co-exist durably and support the replication of a diversity of related viruses, consequently maintaining the host-virus system as specific host strains peak and collapse. Another hypothesis to explain the maintenance of the viral diversity would be the need for different virus lineages to co-infect the same host cells in a cooperative manner, that is, each virus contributes positively to the replication of the entire viral pool. For instance, this occurs in RNA virus quasispecies through genetic complementation when two or more variants carrying deleterious mutations at different loci share their gene products to compensate for these defects, thus restoring normal functions (64, 65). However, the diversity of phycodnaviruses cannot be adequately explained by the viral quasispecies model, although certain similarities are apparent. RNA quasispecies involve a high copy number of genome variants arising from the high mutation rates of RNA viruses which all infect the same host (66). This phenomenon produces so-called “mutant swarms” that foster unique dynamics between viruses where variants are acting (66–68) in competition among themselves. The results of this, to the virus-susceptible host(s)’ detriment and the virus population’s benefit, is the production of a “genome repertoire” in advance of a host’s response, or other factors that could affect the host-virus dynamics of a system in the future (67). However, instead of multiple viral, quasi-identical (but still genetically distinguishable) strain-like species of the RNA quasispecies, HRAP phycodnaviruses exhibited substantial genomic sequence divergence given dotplot alignments of contigs containing a single-copy PolB gene (Fig. S13). RNA viruses are capable of evolving as quasispecies because of their error-prone (i.e.*,* low fidelity) RNA polymerase, resulting in a mutational rate up to 1 million times that of their hosts (69). *Nucleocytoviricota* does not feature the same high rate of mutation, largely attributed to high-fidelity PolB repair machinery (70, 71). Thus, it seems likely that the phycodnaviral lineages had begun to diverge well before they entered the culture basin and on a larger time scale than between members of a typical RNA quasispecies. Within RNA quasispecies*,* diverse genotypes are permissible to handle the diversity of strains occurring within their host target species, such as the bloom species *Heterosigma akashiwo,* which has a wide distribution (72). An array of virus strains is kept as a repertoire of infectious agents to handle the dynamics of their host strains. It may not be the case that new algal strains are developing, but rather a dance between dominant strains, and strains that are simply maintained, which changes over time or space. Although the exact mechanism is not the same between the HRAP phycodnaviruses and the RNA viruses, the pressure put on the viruses to evolve (i.e., the maintenance of specific traits of a species pangenome) and the evolutionary response, or “solution,” are similar. The said pressure is the dynamics and repertoire of microalgae strains and closely related species (in our case potentially the strains of a *Picochlorum* sp.), and the solution is having a complementary genomic repertoire of related viruses at hand; in other words, a genomic repertoire (pangenome) to handle host diversity similar to RNA quasispecies evolutionary “behavior.”

Closely related viruses that infect the same host are typically classified in the same species. Although the phycodnaviruses in this study are phylogenetically related and potentially infect the same host, their genomes may be too divergent to be recognized as belonging to the same species. Alga strains within one alga species can be virus susceptible or resistant (e.g., within the system of *Aureocococcus anophagefferens* and *Kratosvirus quantuckense* [73–76]), thereby a dense culture with different strains could support a group of closely related viruses that are capable of infecting some (one or more) strains of an alga species and not others. Although the concept of several different viruses infecting one species is not new, it is specifically intriguing that the maintenance of several phycodnaviruses could reflect the persistence of algal strains within possibly one host species. Indeed, studied coccolithoviruses (e.g., EhV, also a member of *Phycodnaviridae*) of the bloom-forming algae, *Emiliania huxleyi* have shown similar results to ours. These studies have suggested that a highly dynamic virus population (including virus variation reflecting the water column) existed with a stable population of *E. huxleyi* (77). A separate study also showed EhV diversity was lower in enclosures with elevated *p*CO_2_ compared to “ambient” *p*CO_2_ treatments (although the authors caution that it is unclear if this is a direct impact of the *p*CO_2_ on the viruses or not), suggesting that ocean acidification may reduce the diversity of viruses important for controlling algal blooms (78). Of great relevance was another study of EhV that focused on MCP diversity using a ~238 bp amplicon (79). This study revealed a collection of 151 unique EhV sequences across >6,000 km of open ocean, which were clustered by distinct sampling locations (using principal component analysis). These sequences included MCP representatives which appeared multiple times and several a single time (*n* = 34), which the authors concluded did not fully represent the viral richness of their sampling sites (by rarefraction analysis). Consequently, elevating these results from an interesting evolutionary phenomenon to ties with understanding the impacts of ocean acidification on viral diversity and in turn how this may impact bloom cycles in our changing climate.

Of importance is to note that recording viral diversity such as within this study (specifically “group d,” Fig. 3) does not verify an infectious particle—a caveat discussed by the authors of the open ocean MCP study mentioned above (79). In the context, we have recorded many viruses correlated with a *Picochlorum* species; however, in theory, these could be non-functional viruses produced by lysing *Picochlorum* and captured by our sequencing. Within our study, we did see the reappearance of all *Phycodnaviridae* tracked MCPs from “group d” increasing the likelihood that these viruses are achieving replication in their host, with the seemingly unlikely alternative being that identical non-functioning viruses are produced three separate times within approximately one month and maintained for 20 + days in culture.

### Viruses may be a factor in mass mortality and bloom control within the HRAP

Several culture crashes occurred in the HRAP throughout 2017 and 2018, including during periods when our data set was not at a high enough resolution to make implications about whether viruses were a significant factor or not. For example, *Mimiviridae* 2 (Fig. 3G) and PLV 8 (Fig. 4H) are the only viruses tracked that seemingly persisted before the July 2018 crash (excluding 2017 data), although not many samples were collected near the July 2018 crash. The resolution around the three crashes occurring in Autumn 2018 is quite well defined overall, and consequently, the abundance of viruses (Fig. 3 and 4) appears to coincide well with these crashes, thereby we hypothesize that the group of mostly phycodnaviruses is contributing to the crash of primarily *Picochlorum*. This is not unusual as viruses are known to participate in bloom termination (80–83). In the context of the co-occurring PLVs, it is possible they are simply present and absent based on the presence and the removal of their potential hosts (e.g., introduced by other predators, parasites, or fluctuating seasonal conditions) and the overall crash and re-initiation of the system, but more significantly we do not know the effect of these PLVs on their hosts and if PLVs can contribute to or cause bloom termination in marine environments (e.g., if these PLVs lyse their hosts versus behave like virophages with a tripartite infection system and genomic integration [17]). Broadly speaking, the HRAP may not completely reflect the trajectory of Mediterranean Sea sourced water used to initiate the HRAP culture, given that some algae only appear around times of culture restarts and then disappear after or are not maintained long in the HRAP (*Picochlorum* 3, *Chlorella*, and *Dictyosphaerium*; Fig. 5D); however, for the microalgae that persist and the viruses occurring alongside there appears to be an algal bloom like maintenance and resulting virus bloom termination.

### Conclusion

Overall, this study demonstrates the incredible diversity and dynamics of DNA viruses within an HRAP. Ultimately, we suggest that the HRAP environment can mimic a marine microalgae bloom. This resulting diversity of putative *Phycodnaviridae* contains a repertoire of viruses available to infect, at least one genus (and potentially the strains of one or more species) of microalgae. Although the exact progression to reach this result is different than that of RNA quasispecies, we cannot deny similarities in the end results of this potential virus-host dynamic (i.e., genetically similar virus variants infecting a single host). Ultimately, it appears that viruses could be meaningfully contributing to the termination of an algal bloom in the HRAP system which will continue to grow in its importance given our changing climate, and more specifically changing oceans.

## Data Availability

Raw sequences were deposited at the National Center for Biotechnology Information (NCBI) under the BioProject identifiers PRJNA1098217 for 18S rDNA sequencing and PRJNA751746 for Illumina MiSeq sequencing. Illumina HiSeq sequences generated by the U.S. Joint Genome Institute are available on the JGI genomics portal under the identifier 504991 (https://genome.jgi.doe.gov/portal/).

## SUPPLEMENTAL MATERIAL

Figure S1 and S2 mbio.02803-24-s0001.docx FACS by SSC and FITC on viral populations and broad taxonomy of contigs.

Figure S3 mbio.02803-24-s0002.docx PLV phylogeny.

Figure S4 and S5 mbio.02803-24-s0003.docx Virophage phylogeny and annotation of viral ORFs.

Figure S6 mbio.02803-24-s0004.docx Phylogenetic reconstruction of 7 AMGs recovered from giant viruses.

Figure S7 mbio.02803-24-s0005.docx PCA of giant viruses, a virophage, and their putative microeukaryotic hosts.

Figure S8 and S9 mbio.02803-24-s0006.docx SIMPROF clustering.

Supplementary Tables and Data files mbio.02803-24-s0007.xlsx Tables S1-S6 and Data Files S1 and S2.

Figure S10 and S11 mbio.02803-24-s0008.docx PCA and SIMPROF and giant virus phylogeny (PolB).

Figure S12 mbio.02803-24-s0009.docx Giant virus phylogeny (ATPase).

Figure S13 and captions mbio.02803-24-s0010.docx Dotplot alignment of giant virus (*Phycodnaviridae*) PolB genes and captions for supplemental tables and data files.

ASM does not own the copyrights to Supplemental Material that may be linked to, or accessed through, an article. The authors have granted ASM a non-exclusive, world-wide license to publish the Supplemental Material files. Please contact the corresponding author directly for reuse.
