# Supplementary material for: Viral dynamics in a high-rate algal pond reveals a burst of Phycodnaviridae diversity correlated with episodic algal mortality
Source: mBio. 2024 Nov 12;15(12):e02803-24. doi: 10.1128/mbio.02803-24 (PMC11633385; doi:10.1128/mbio.02803-24)
Supplement: Figure S3 — PLV phylogeny. [file mbio.02803-24-s0002.docx]

SUPPLEMENTAL ONLINE INFORMATION

For publication in conjunction with the following:

Viral dynamics in a high rate algal pond reveals a burst of *Phycodnaviridae* diversity correlated with episodic algal mortality

Chase EE^1,2,3^, Pitot T^4^, Bouchard S^1^, Triplet S^5^, Przybyla C^5^, Gobet A^5^, Desnues C^1,2^, and Blanc G^1^.

*^1^ Microbiologie Environnementale Biotechnologie, Institut Méditerranéen d'Océanologie, Campus de Luminy, 163 Avenue de Luminy, 13009 Marseille, France*

*^2^ Institut hospitalo-universitaire (IHU) Méditerranée infection, 19-21 Boulevard Jean Moulin, 13005 Marseille, France*

*^3^ University of Tennessee Knoxville, Department of Microbiology, Ken and Blaire Mossman Bldg, 1311 Cumberland Ave #307, Knoxville, TN 37996*

*^4^ Department of Biochemistry, Microbiology and Bioinformatics, Université Laval, 2325 rue de l’Université, Québec, QC G1V0A6, Canada*

*^5^ MARBEC, Univ Montpellier, CNRS, Ifremer, IRD, Sète, France*

**SUPPLEMENTAL FIGURES**


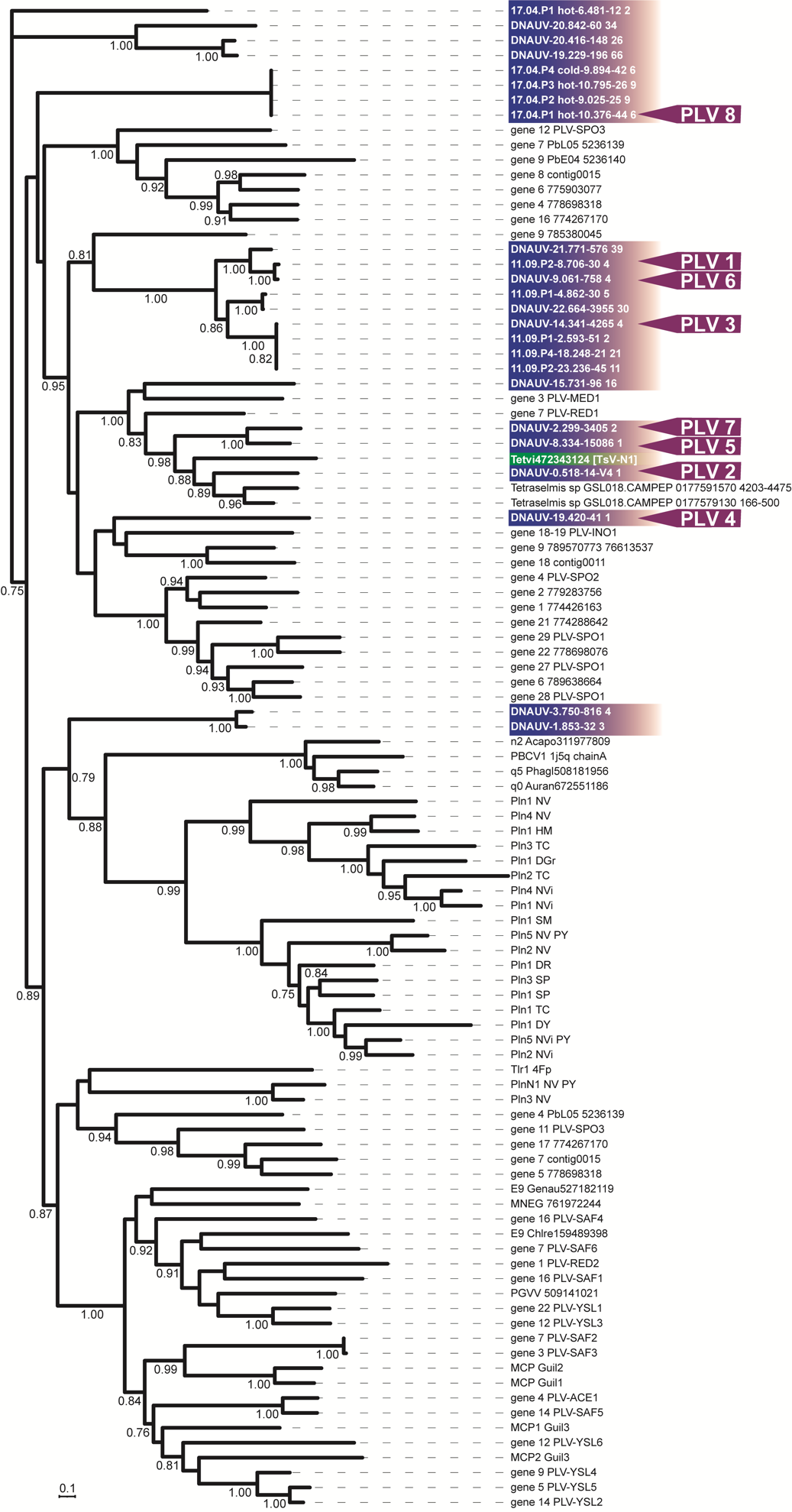


**Figure S3.** Putative polinton-like viruses (PLVs) recovered from HRAP metagenomes (indicated in red). The PLV TsV-N1 is indicated and the green line covers the PLVs that are clustering with it phylogenetically. The phylogeny based on the MCP gene. PLVs used in qPCR tracking are indicated in blue. PLV 8 is excluded due to the sequence dissimilarity relative to the rest of the tracked PLVs. Bootstrap values above 0.70 are displayed. All MCP (excluding those in red) were extracted from public databases (see Chapter 3 for more detailed methods).
