# Supplementary material for: Viral dynamics in a high-rate algal pond reveals a burst of Phycodnaviridae diversity correlated with episodic algal mortality
Source: mBio. 2024 Nov 12;15(12):e02803-24. doi: 10.1128/mbio.02803-24 (PMC11633385; doi:10.1128/mbio.02803-24)
Supplement: Figure S4 and S5 — Virophage phylogeny and annotation of viral ORFs. [file mbio.02803-24-s0003.docx]

SUPPLEMENTAL ONLINE INFORMATION

For publication in conjunction with the following:

Viral dynamics in a high rate algal pond reveals a burst of *Phycodnaviridae* diversity correlated with episodic algal mortality

Chase EE^1,2,3^, Pitot T^4^, Bouchard S^1^, Triplet S^5^, Przybyla C^5^, Gobet A^5^, Desnues C^1,2^, and Blanc G^1^.

*^1^ Microbiologie Environnementale Biotechnologie, Institut Méditerranéen d'Océanologie, Campus de Luminy, 163 Avenue de Luminy, 13009 Marseille, France*

*^2^ Institut hospitalo-universitaire (IHU) Méditerranée infection, 19-21 Boulevard Jean Moulin, 13005 Marseille, France*

*^3^ University of Tennessee Knoxville, Department of Microbiology, Ken and Blaire Mossman Bldg, 1311 Cumberland Ave #307, Knoxville, TN 37996*

*^4^ Department of Biochemistry, Microbiology and Bioinformatics, Université Laval, 2325 rue de l’Université, Québec, QC G1V0A6, Canada*

*^5^ MARBEC, Univ Montpellier, CNRS, Ifremer, IRD, Sète, France*

**SUPPLEMENTAL FIGURES**


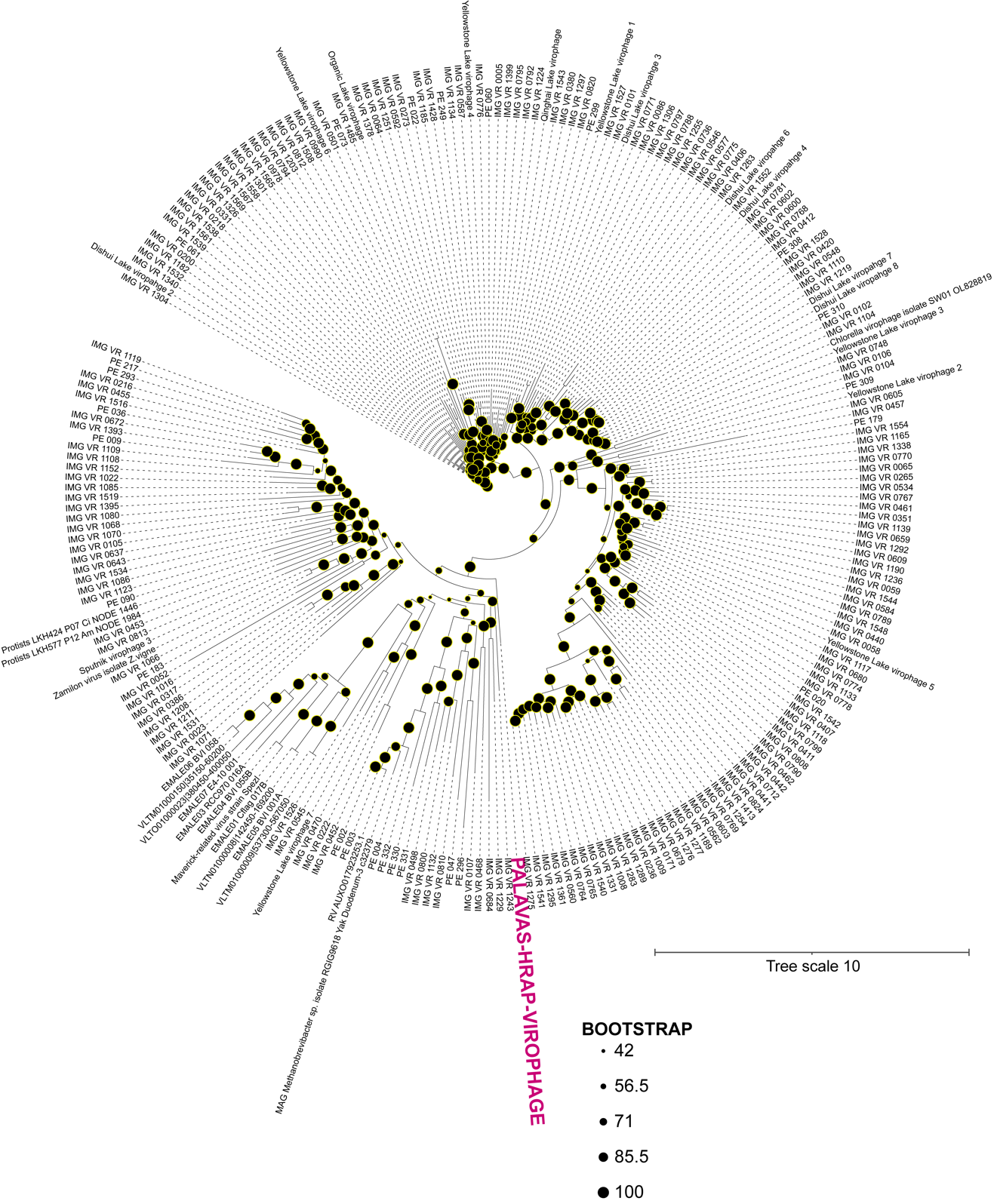


**Figure S4.** Putative virophage (highlighted with pink) assembled from HRAP assemblies. Phylogeny is based on major capsid protein sequenced retrieved from IMG/VR v4. Bootstrap probabilities (1000 replicates) are displayed.


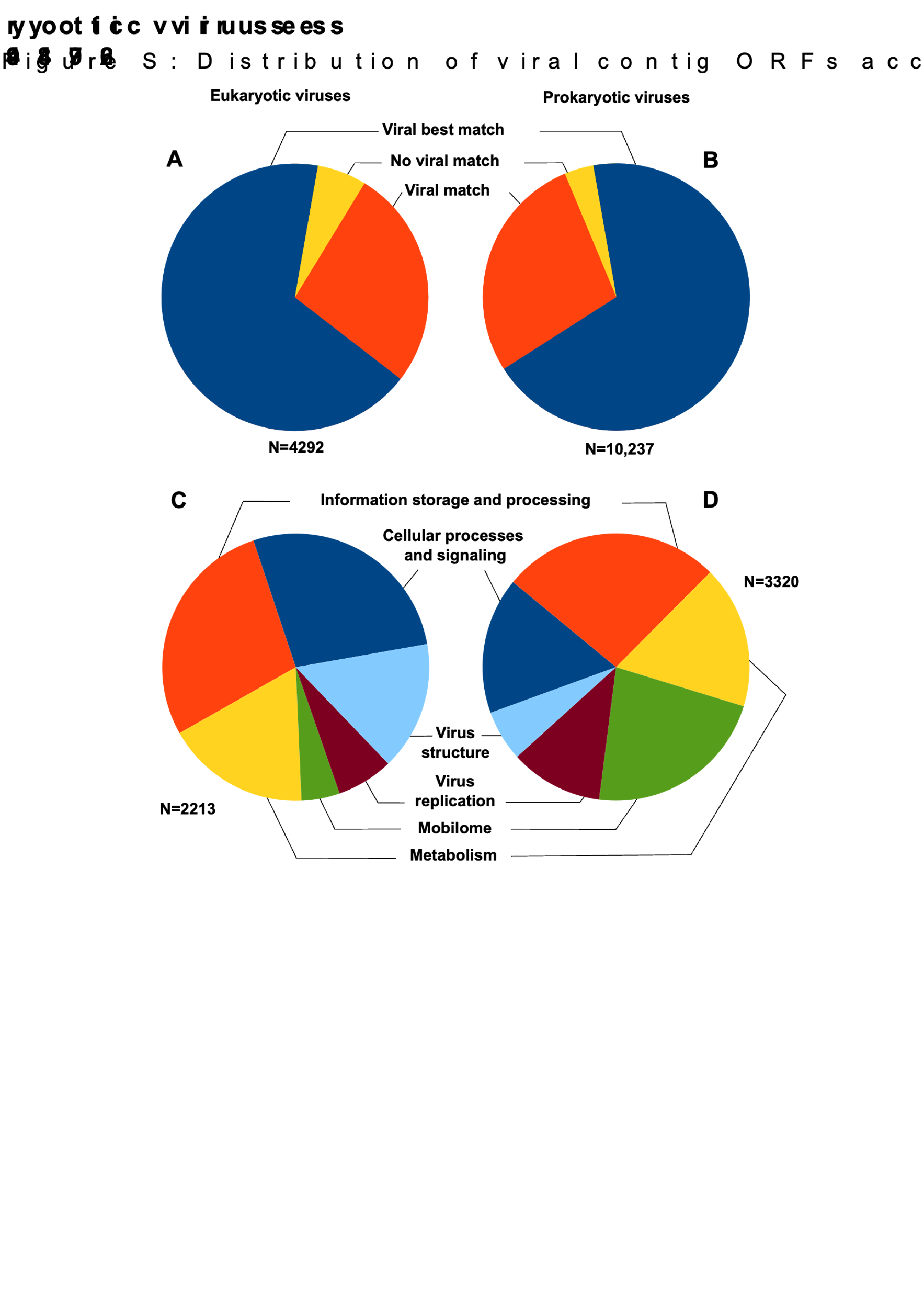


**Figure S5.** Annotation of viral contig ORFs. (**A, B**) Distribution of ORFs according to their match pattern in Uniref100 for the eukaryotic and prokaryotic contigs respectively. "Viral best match" designates ORFs whose top hit is of viral origin (MMSEQS search against Uniref100). "Viral match" refers to ORFs that do not have a viral top hit but still produce significant MMSEQS alignments with viral proteins. "No viral match" indicates ORFs that have only cellular matches in Uniref100 using an e-value<1E-5. (**C, D**) Broad functional categories of viral contig ORFs based on the analysis of COG and VOG best matches for the eukaryotic and prokaryotic contigs, respectively.
