# Supplementary material for: Viral dynamics in a high-rate algal pond reveals a burst of Phycodnaviridae diversity correlated with episodic algal mortality
Source: mBio. 2024 Nov 12;15(12):e02803-24. doi: 10.1128/mbio.02803-24 (PMC11633385; doi:10.1128/mbio.02803-24)
Supplement: Figure S6 — Phylogenetic reconstruction of 7 AMGs recovered from giant viruses. [file mbio.02803-24-s0004.docx]

SUPPLEMENTAL ONLINE INFORMATION

For publication in conjunction with the following:

Viral dynamics in a high rate algal pond reveals a burst of *Phycodnaviridae* diversity correlated with episodic algal mortality

Chase EE^1,2,3^, Pitot T^4^, Bouchard S^1^, Triplet S^5^, Przybyla C^5^, Gobet A^5^, Desnues C^1,2^, and Blanc G^1^.

*^1^ Microbiologie Environnementale Biotechnologie, Institut Méditerranéen d'Océanologie, Campus de Luminy, 163 Avenue de Luminy, 13009 Marseille, France*

*^2^ Institut hospitalo-universitaire (IHU) Méditerranée infection, 19-21 Boulevard Jean Moulin, 13005 Marseille, France*

*^3^ University of Tennessee Knoxville, Department of Microbiology, Ken and Blaire Mossman Bldg, 1311 Cumberland Ave #307, Knoxville, TN 37996*

*^4^ Department of Biochemistry, Microbiology and Bioinformatics, Université Laval, 2325 rue de l’Université, Québec, QC G1V0A6, Canada*

*^5^ MARBEC, Univ Montpellier, CNRS, Ifremer, IRD, Sète, France*

**Figure S6**. Phylogenetic trees of viral proteins with putative HGT origin. Phylogenetic reconstruction was carried out following the general procedure provided in the Material and Methods section. Scale bar reflects the average number of amino acid substitutions per site. The numbers beside the nodes represent the proportion of bootstrap support. The protein families studied are (**A**) cobalt transporter, (**B**) iron transporter, (**C**) Calvin cycle protein CP12, (**D**) Heme oxygenase 1, (**E**) phycocyanobilin:ferredoxin oxidoreductase, (**F**) photosystem 1 assembly protein Ycf3 and (**G**) phosphatidylserine decarboxylase.
