# Supplementary material for: Viral dynamics in a high-rate algal pond reveals a burst of Phycodnaviridae diversity correlated with episodic algal mortality
Source: mBio. 2024 Nov 12;15(12):e02803-24. doi: 10.1128/mbio.02803-24 (PMC11633385; doi:10.1128/mbio.02803-24)
Supplement: Figure S7 — PCA of giant viruses, a virophage, and their putative microeukaryotic hosts. [file mbio.02803-24-s0005.docx]

SUPPLEMENTAL ONLINE INFORMATION

For publication in conjunction with the following:

Viral dynamics in a high rate algal pond reveals a burst of *Phycodnaviridae* diversity correlated with episodic algal mortality

Chase EE^1,2,3^, Pitot T^4^, Bouchard S^1^, Triplet S^5^, Przybyla C^5^, Gobet A^5^, Desnues C^1,2^, and Blanc G^1^.

*^1^ Microbiologie Environnementale Biotechnologie, Institut Méditerranéen d'Océanologie, Campus de Luminy, 163 Avenue de Luminy, 13009 Marseille, France*

*^2^ Institut hospitalo-universitaire (IHU) Méditerranée infection, 19-21 Boulevard Jean Moulin, 13005 Marseille, France*

*^3^ University of Tennessee Knoxville, Department of Microbiology, Ken and Blaire Mossman Bldg, 1311 Cumberland Ave #307, Knoxville, TN 37996*

*^4^ Department of Biochemistry, Microbiology and Bioinformatics, Université Laval, 2325 rue de l’Université, Québec, QC G1V0A6, Canada*

*^5^ MARBEC, Univ Montpellier, CNRS, Ifremer, IRD, Sète, France*

**SUPPLEMENTAL FIGURES**


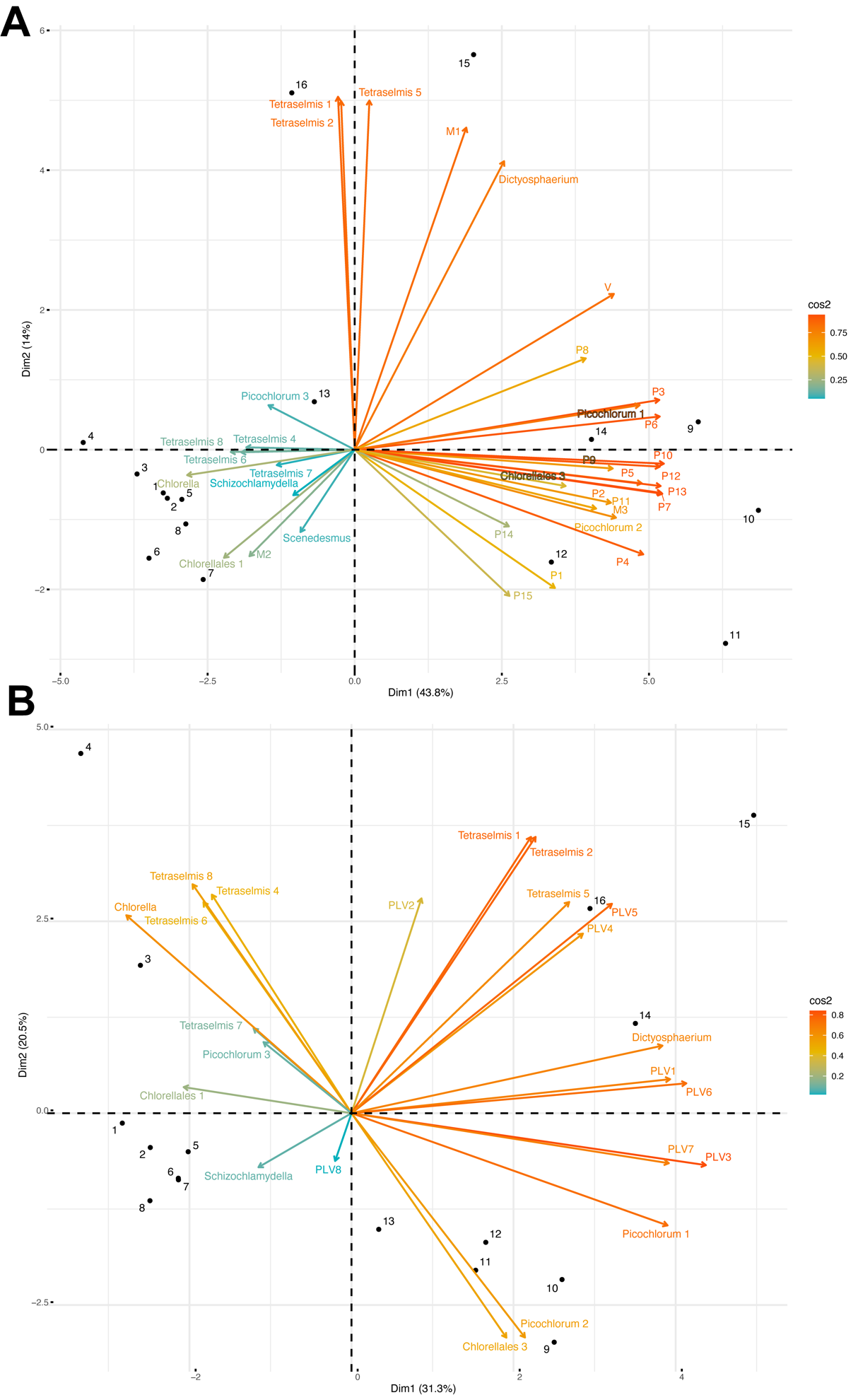


**Figure S7.** Principal component analyses using combined and normalised qPCR data and 18S rDNA ASVs (*i.e.,* metabarcoding) on tracked putative viruses and potential alga (Chlorophyta) hosts for **(A)** *Mimiviridae* (M), *Phycodnaviridae* (P), and virophage (V), and **(B)** polinton-like viruses (PLV). Cos2 reports the strength of the principal component for the observations (i.e. virus or potential hosts), where a higher value depicts a stronger relationship between them or a “good representation”. The vector length of each observations represents the contribution they make to the ordination. Dates are represented by numbered objects, where 2017 is composed of 1–5 (April is 1 and 2, May is 3, June is 4 and 5), and 2018 is composed of 6–16 (April is 6, May is 7–9, August is 9, September Is 10–13, and October is 14–16).
