# Supplementary material for: Viral dynamics in a high-rate algal pond reveals a burst of Phycodnaviridae diversity correlated with episodic algal mortality
Source: mBio. 2024 Nov 12;15(12):e02803-24. doi: 10.1128/mbio.02803-24 (PMC11633385; doi:10.1128/mbio.02803-24)
Supplement: Figure S8 and S9 — SIMPROF clustering. [file mbio.02803-24-s0006.docx]

SUPPLEMENTAL ONLINE INFORMATION

For publication in conjunction with the following:

Viral dynamics in a high rate algal pond reveals a burst of *Phycodnaviridae* diversity correlated with episodic algal mortality

Chase EE^1,2,3^, Pitot T^4^, Bouchard S^1^, Triplet S^5^, Przybyla C^5^, Gobet A^5^, Desnues C^1,2^, and Blanc G^1^.

*^1^ Microbiologie Environnementale Biotechnologie, Institut Méditerranéen d'Océanologie, Campus de Luminy, 163 Avenue de Luminy, 13009 Marseille, France*

*^2^ Institut hospitalo-universitaire (IHU) Méditerranée infection, 19-21 Boulevard Jean Moulin, 13005 Marseille, France*

*^3^ University of Tennessee Knoxville, Department of Microbiology, Ken and Blaire Mossman Bldg, 1311 Cumberland Ave #307, Knoxville, TN 37996*

*^4^ Department of Biochemistry, Microbiology and Bioinformatics, Université Laval, 2325 rue de l’Université, Québec, QC G1V0A6, Canada*

*^5^ MARBEC, Univ Montpellier, CNRS, Ifremer, IRD, Sète, France*

**SUPPLEMENTAL FIGURES**


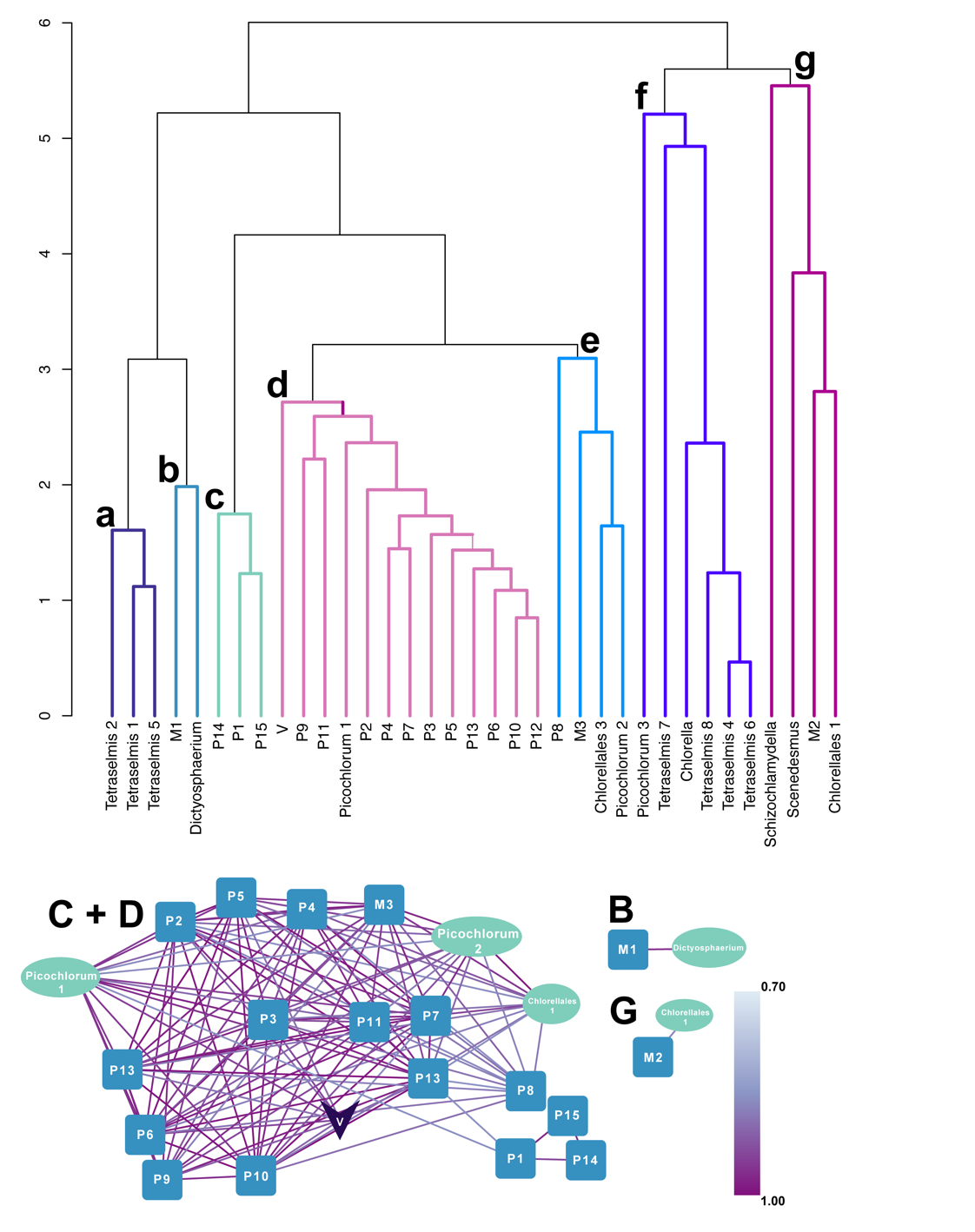


**Figure S8.** Hierarchical clustering (SIMPROF, α = 0.1) of potential hosts (Chlorophyta) and viruses of interest; *Phycodnaviridae* (P), *Mimiviridae* (M), and virophage (V) with overlapping data among 18S rDNA host and virus qPCR results. Predicted groupings are depicted **(a–g)** using a network visualisation. Darker colours represented a higher correlation between group members, where 1 is an exact correlation and 0 is no correlation.


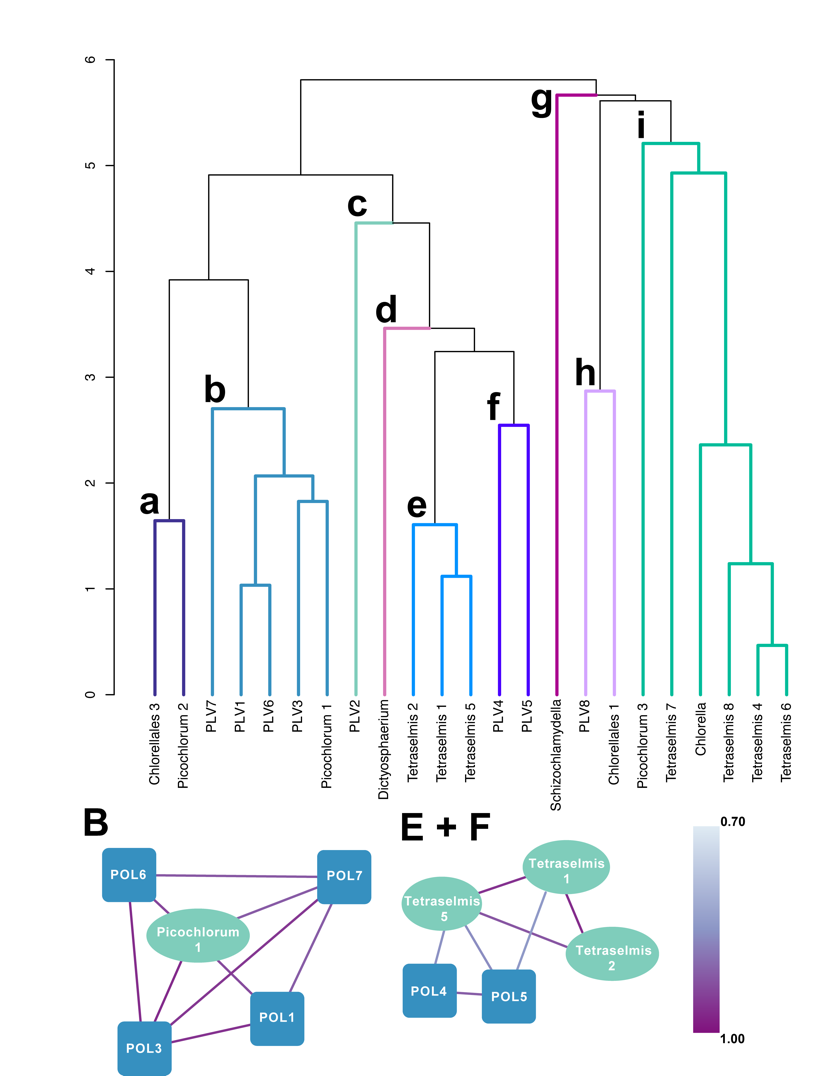


**Figure S9.** Hierarchical clustering (SIMPROF, α = 0.1) of potential hosts (Chlorophyta) and viruses of interest; polinton-like viruses (PLV). Predicted groupings are depicted **(a–i)** using a network visualisation. Darker colours represented a higher correlation between group members, where 1 is an exact correlation and 0 is no correlation.
