# Supplementary material for: Viral dynamics in a high-rate algal pond reveals a burst of Phycodnaviridae diversity correlated with episodic algal mortality
Source: mBio. 2024 Nov 12;15(12):e02803-24. doi: 10.1128/mbio.02803-24 (PMC11633385; doi:10.1128/mbio.02803-24)
Supplement: Figure S10 and S11 — PCA and SIMPROF and giant virus phylogeny (PolB). [file mbio.02803-24-s0008.docx]

SUPPLEMENTAL ONLINE INFORMATION

For publication in conjunction with the following:

Viral dynamics in a high rate algal pond reveals a burst of *Phycodnaviridae* diversity correlated with episodic algal mortality

Chase EE^1,2,3^, Pitot T^4^, Bouchard S^1^, Triplet S^5^, Przybyla C^5^, Gobet A^5^, Desnues C^1,2^, and Blanc G^1^.

*^1^ Microbiologie Environnementale Biotechnologie, Institut Méditerranéen d'Océanologie, Campus de Luminy, 163 Avenue de Luminy, 13009 Marseille, France*

*^2^ Institut hospitalo-universitaire (IHU) Méditerranée infection, 19-21 Boulevard Jean Moulin, 13005 Marseille, France*

*^3^ University of Tennessee Knoxville, Department of Microbiology, Ken and Blaire Mossman Bldg, 1311 Cumberland Ave #307, Knoxville, TN 37996*

*^4^ Department of Biochemistry, Microbiology and Bioinformatics, Université Laval, 2325 rue de l’Université, Québec, QC G1V0A6, Canada*

*^5^ MARBEC, Univ Montpellier, CNRS, Ifremer, IRD, Sète, France*

**SUPPLEMENTAL FIGURES**


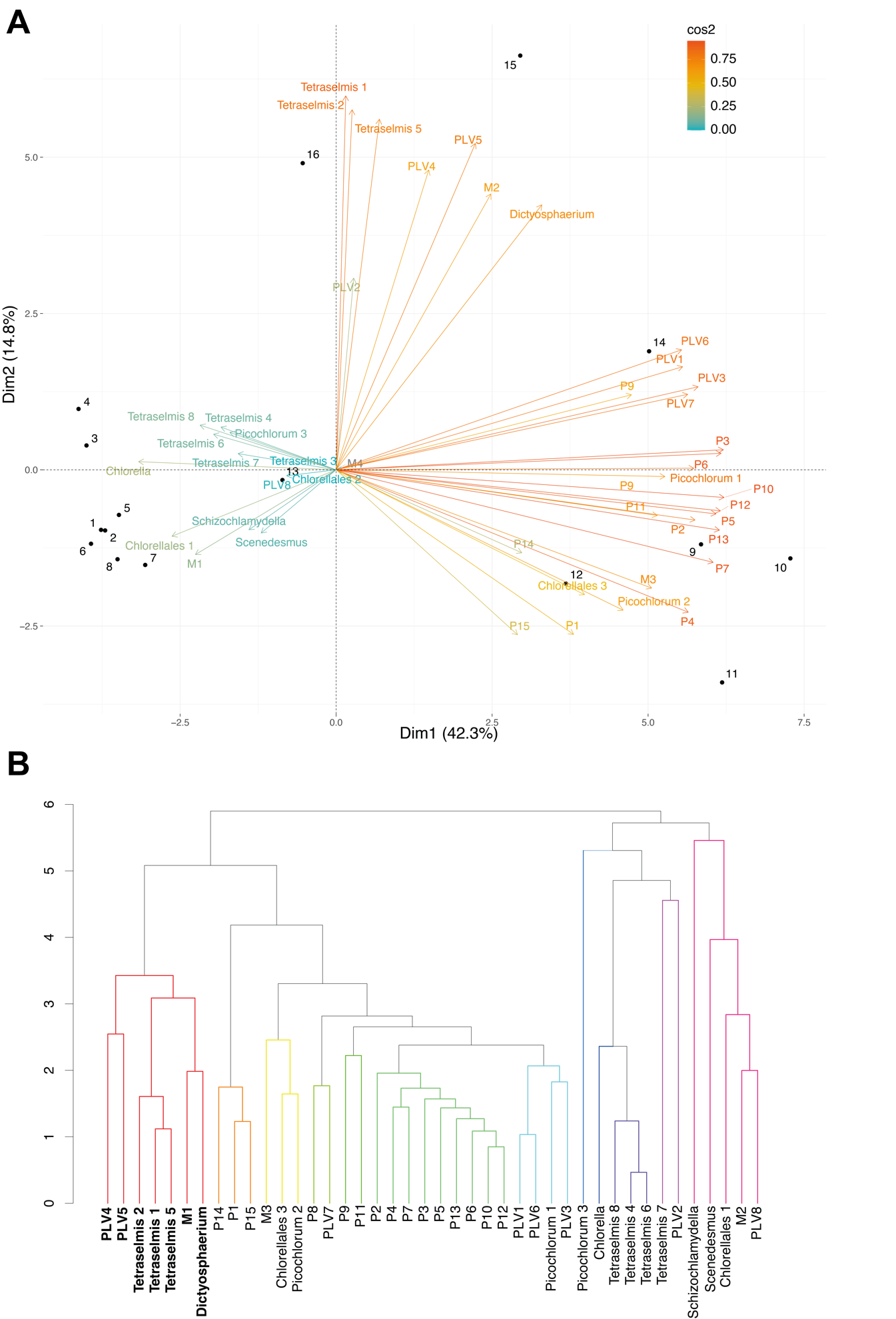


**Figure S10.** Investigation of *Nucleocytoviricota* and PLV relationships through **(A)** principal component analyses using combined and normalised qPCR data and 18S rDNA ASVs (*i.e.,* metabarcoding) on tracked putative viruses and potential alga (Chlorophyta) hosts for *Mimiviridae* (M), *Phycodnaviridae* (P), and polinton-like viruses (PLV). Cos2 reports the strength of the principal component for the observations (*i.e.* virus or potential hosts), where a higher value depicts a stronger relationship between them or a “good representation”. The vector length of each observations represents the contribution they make to the ordination. Dates are represented by numbered objects, where 2017 is composed of 1–5 (April is 1 and 2, May is 3, June is 4 and 5), and 2018 is composed of 6–16 (April is 6, May is 7–9, August is 9, September Is 10–13, and October is 14–16). A second analysis: **(B**) Hierarchical clustering (SIMPROF, α = 0.1) of potential hosts (Chlorophyta) and viruses of interest, where colours represent different groups clustering at our minimum. Bolded text signifies hosts and viruses mentioned within the manuscript.


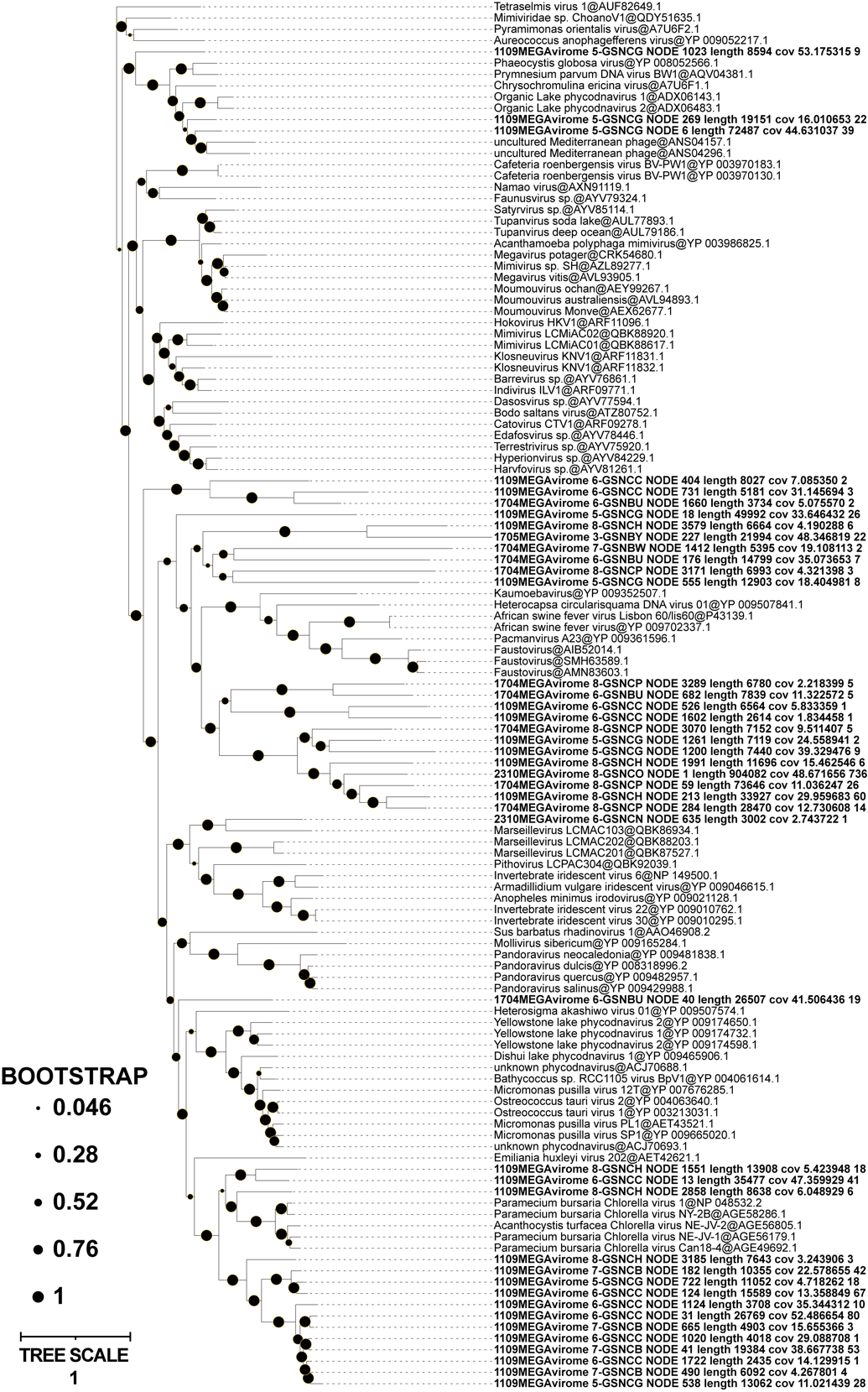


**Figure S11.** Putative Nucleocytoviricota phylogeny based on the DNA polymerase B gene (PolB). Reference sequences were downloaded by NCBI GenBank (accession numbers as indicated) and sequenced recovered from the HRAP are included (in bold). Bootstrap values are indicated by circle size.
