# Supplementary material for: Viral dynamics in a high-rate algal pond reveals a burst of Phycodnaviridae diversity correlated with episodic algal mortality
Source: mBio. 2024 Nov 12;15(12):e02803-24. doi: 10.1128/mbio.02803-24 (PMC11633385; doi:10.1128/mbio.02803-24)
Supplement: Figure S12 — Giant virus phylogeny (ATPase). [file mbio.02803-24-s0009.docx]

SUPPLEMENTAL ONLINE INFORMATION

For publication in conjunction with the following:

Viral dynamics in a high rate algal pond reveals a burst of *Phycodnaviridae* diversity correlated with episodic algal mortality

Chase EE^1,2,3^, Pitot T^4^, Bouchard S^1^, Triplet S^5^, Przybyla C^5^, Gobet A^5^, Desnues C^1,2^, and Blanc G^1^.

*^1^ Microbiologie Environnementale Biotechnologie, Institut Méditerranéen d'Océanologie, Campus de Luminy, 163 Avenue de Luminy, 13009 Marseille, France*

*^2^ Institut hospitalo-universitaire (IHU) Méditerranée infection, 19-21 Boulevard Jean Moulin, 13005 Marseille, France*

*^3^ University of Tennessee Knoxville, Department of Microbiology, Ken and Blaire Mossman Bldg, 1311 Cumberland Ave #307, Knoxville, TN 37996*

*^4^ Department of Biochemistry, Microbiology and Bioinformatics, Université Laval, 2325 rue de l’Université, Québec, QC G1V0A6, Canada*

*^5^ MARBEC, Univ Montpellier, CNRS, Ifremer, IRD, Sète, France*

**SUPPLEMENTAL FIGURES**


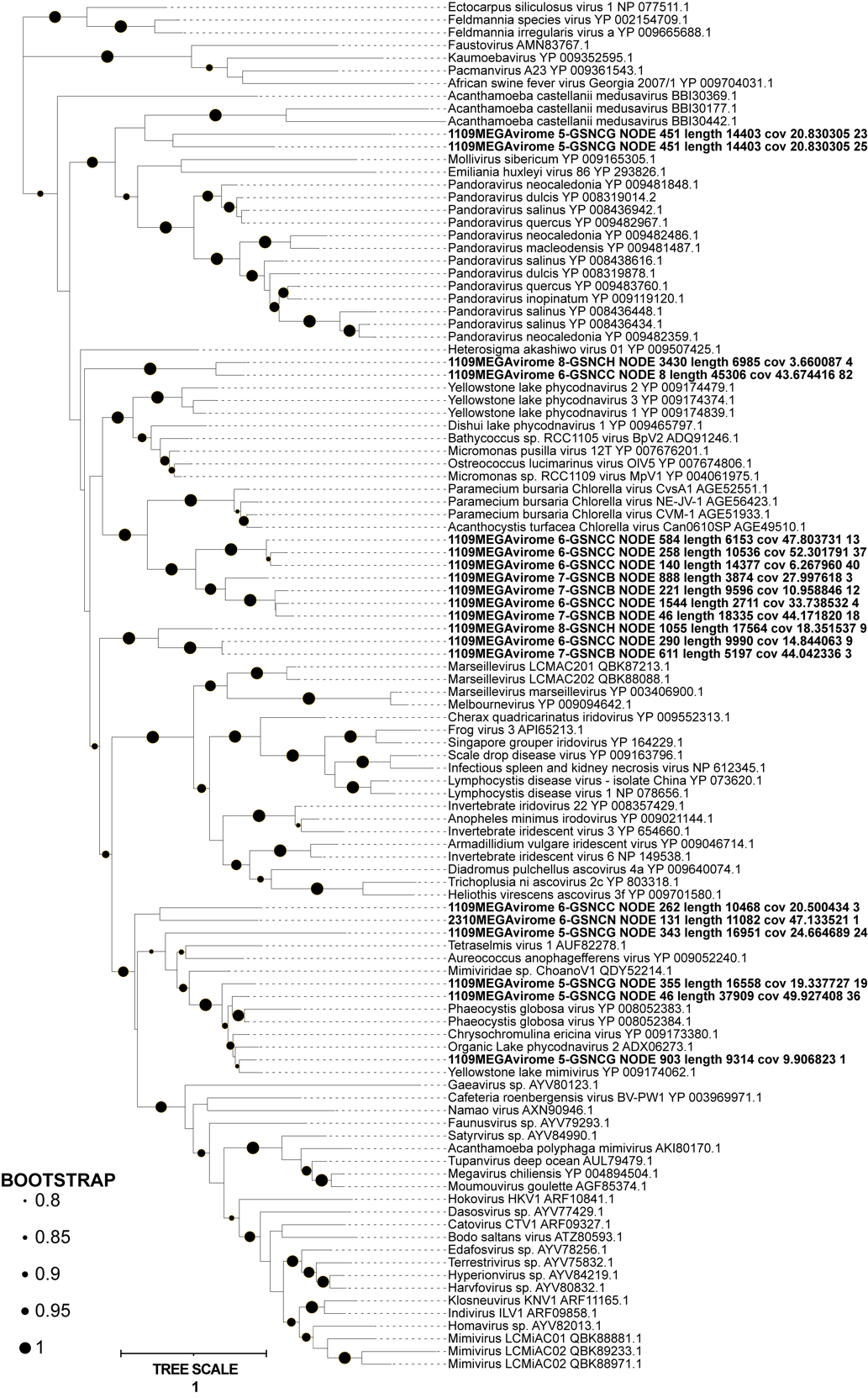


**Figure S12.** Putative Nucleocytoviricota phylogeny based on the ATPase gene. Reference sequences were downloaded by NCBI GenBank (accession numbers as indicated) and sequenced recovered from the HRAP are included (in bold). Bootstrap values are indicated by circle size.
