# Supplementary material for: Viral dynamics in a high-rate algal pond reveals a burst of Phycodnaviridae diversity correlated with episodic algal mortality
Source: mBio. 2024 Nov 12;15(12):e02803-24. doi: 10.1128/mbio.02803-24 (PMC11633385; doi:10.1128/mbio.02803-24)
Supplement: Figure S13 and captions — Dotplot alignment of giant virus (Phycodnaviridae) PolB genes and captions for supplemental tables and data files. [file mbio.02803-24-s0010.docx]

SUPPLEMENTAL ONLINE INFORMATION

For publication in conjunction with the following:

Viral dynamics in a high rate algal pond reveals a burst of *Phycodnaviridae* diversity correlated with episodic algal mortality

Chase EE^1,2,3^, Pitot T^4^, Bouchard S^1^, Triplet S^5^, Przybyla C^5^, Gobet A^5^, Desnues C^1,2^, and Blanc G^1^.

*^1^ Microbiologie Environnementale Biotechnologie, Institut Méditerranéen d'Océanologie, Campus de Luminy, 163 Avenue de Luminy, 13009 Marseille, France*

*^2^ Institut hospitalo-universitaire (IHU) Méditerranée infection, 19-21 Boulevard Jean Moulin, 13005 Marseille, France*

*^3^ University of Tennessee Knoxville, Department of Microbiology, Ken and Blaire Mossman Bldg, 1311 Cumberland Ave #307, Knoxville, TN 37996*

*^4^ Department of Biochemistry, Microbiology and Bioinformatics, Université Laval, 2325 rue de l’Université, Québec, QC G1V0A6, Canada*

*^5^ MARBEC, Univ Montpellier, CNRS, Ifremer, IRD, Sète, France*

**SUPPLEMENTAL FIGURES**


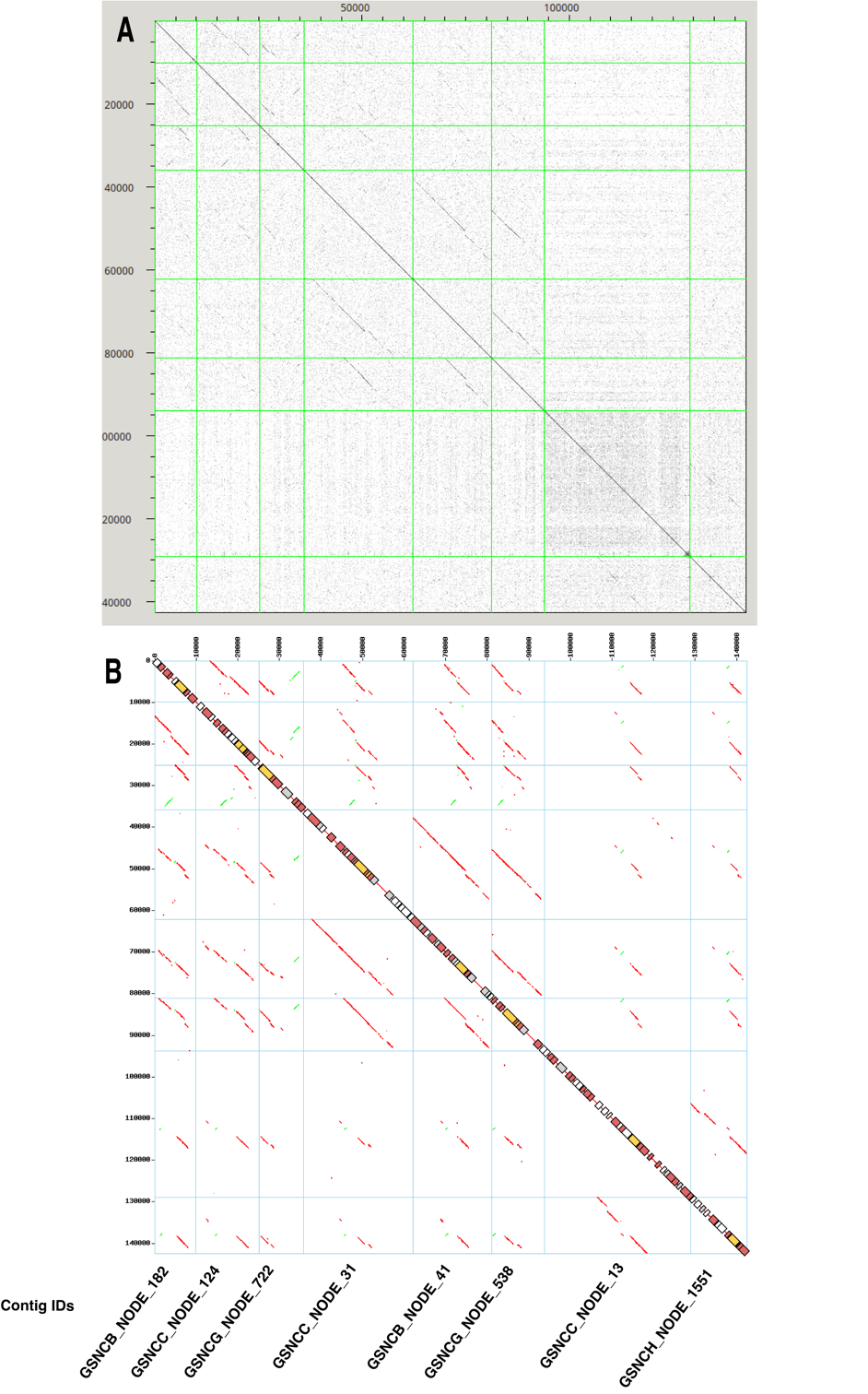


**Figure S13.** Dotplot alignments of *Phycodnaviridae* contigs containing a PolB gene. **(A)** 8 × 8 dotplot alignments of contig nucleotide sequences. **(B)** Dotplot representation of contig’s translated nucleotide sequence matches identified by TBLASTX (red for forward matches, green for reverse matches). Position of the PolB gene on individual contigs is shown by yellow rectangles on the plot diagonal. Additional ORFs with a significant match to viruses or cellular organisms in TrEMBL are shown by red and grey rectangles respectively, and ORFs > 150 codons without match are shown with open rectangles. Contig orders are identical along the x- and y-axis in **A** and **B**. Only contigs greater than 10 kb were used in the analysis. Altogether, a residual gene collinearity is generally observed between *Phycodnaviridae* contigs, with only very few inversions (green matches), however nucleotide sequence conservation is frequently interrupted outside of coding sequences indicating that intergenic sequences have diverged to the point that no significant similarity is yet detectable.

**SUPPLEMENTAL TABLES (CAPTIONS)**

**Table S1.** Selected assembly statistics for ultraviromes and megaviromes based on all contigs over 2000 bp. Analysis conducted using QUAST.

**Table S2.** Contigs taxonomic assignation.

**Table S3.** Putative protein gene (functional) annotation for viral contigs.

**Table S4.** Complete 18S rDNA (V4 variable region) metabarcoding results (*e.g.,* counts and taxonomy) using amplicon sequencing variants (ASV).

**Table S5.** Correlation output of PCA analyses for both the giant virus, virophage, and host portion, the polinton-like viruses and host portion, and giant viruses, polinton-like viruses, and host portion.

**Table S6.** *Picochlorum* putative *Nucleocytoviricota* insertion genes produced by ViralRecall. Number of predicted viral insertions and length are included.

**SUPPLEMENTAL DATA FILES**

**Supplemental Data File 1.** qPCR primer sequences, amplicons, melting temperature, and additional information.

**Supplemental Data File 2.** Phylogeny related files.
